# Catch-bond mechanism of the bacterial adhesin FimH

**DOI:** 10.1038/ncomms10738

**Published:** 2016-03-07

**Authors:** Maximilian M. Sauer, Roman P. Jakob, Jonathan Eras, Sefer Baday, Deniz Eriş, Giulio Navarra, Simon Bernèche, Beat Ernst, Timm Maier, Rudi Glockshuber

**Affiliations:** 1 Institute of Molecular Biology and Biophysics, Department of Biology, ETH, Zurich, Otto-Stern-Weg 5, 8093 Zurich, Switzerland; 2 Biozentrum, University of Basel, Klingelbergstrasse 50/70, 4056 Basel, Switzerland; 3 SIB Swiss Institute of Bioinformatics, University of Basel, Klingelbergstrasse 50/70, 4056 Basel, Switzerland; 4 Institute of Molecular Pharmacy, University of Basel, Klingelbergstrasse 50, 4056 Basel, Switzerland

## Abstract

Ligand–receptor interactions that are reinforced by mechanical stress, so-called catch-bonds, play a major role in cell–cell adhesion. They critically contribute to widespread urinary tract infections by pathogenic *Escherichia coli* strains. These pathogens attach to host epithelia via the adhesin FimH, a two-domain protein at the tip of type I pili recognizing terminal mannoses on epithelial glycoproteins. Here we establish peptide-complemented FimH as a model system for fimbrial FimH function. We reveal a three-state mechanism of FimH catch-bond formation based on crystal structures of all states, kinetic analysis of ligand interaction and molecular dynamics simulations. In the absence of tensile force, the FimH pilin domain allosterically accelerates spontaneous ligand dissociation from the FimH lectin domain by 100,000-fold, resulting in weak affinity. Separation of the FimH domains under stress abolishes allosteric interplay and increases the affinity of the lectin domain. Cell tracking demonstrates that rapid ligand dissociation from FimH supports motility of piliated *E. coli* on mannosylated surfaces in the absence of shear force.

## Abstract (web summary)

Catch bonds have a role in bacterial adhesion and infection by uropathogenic *E. coli.* Here, the authors report crystal structures, molecular dynamics simulations, ligand binding analysis and cell tracking to characterise the catch bond interaction between the adhesin FimH and carbohydrate receptors.

## Graphical abstract

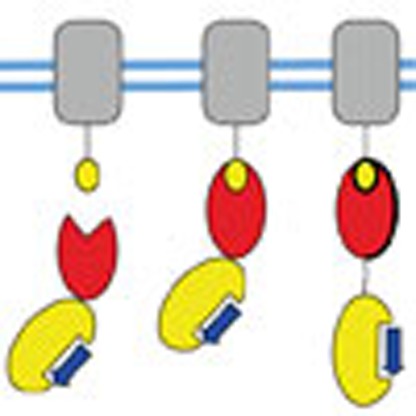

Cell–cell adhesion often occurs under dynamically varying conditions and mechanical stress. In many cell–cell adhesion systems, the lifetime of adhesin–receptor complexes is increased under tensile mechanical force via ‘catch-bonds', which permit capture or retention of cells under flow conditions while still allowing for release under reduced mechanical force. Catch-bond interactions are prominent in vascular systems and are formed, for example, by selectins for leukocyte recruitment^1^,^2^, by cadherins controlling tissue integrity^3^,^4^ in the epithelial adhesion of cancer cells^5^ and by the interactions between T-cell receptors (TCRs) and peptide-bound major histocompatibility complexes (MHC) on antigen-presenting cells^6^,^7^. Catch-bonds also play a major role in bacterial adhesion and infection by uropathogenic *Escherichia coli* strains, which are responsible for the vast majority of urinary tract infections (UTIs) in humans^8^. A first critical step in the establishment of infection is bacterial adhesion to urothelial cells under flow conditions, which is mediated by 0.1−2 μm long, proteinaceous filaments on the bacterial surface termed type 1 pili^9^,^10^. Type 1 pili are composed of up to 3,000 copies of the subunit FimA building the pilus rod, as well as the subunits FimF, FimG and FimH forming the distal tip fibrillum^11^. The adhesin FimH at the fimbrial tip specifically binds in a catch-bond mode^12^ to terminal α-D-linked mannoses of N-linked glycans of the receptor uroplakin 1a on urinary epithelial cells^13^. Owing to its important role in establishing infection, FimH is an attractive target for the development of anti-adhesive drugs for UTI treatment^14^,^15^.

FimH is a two-domain protein, composed of an N-terminal, mannoside-binding lectin domain (FimH_L_) and a C-terminal pilin domain (FimH_P_). FimH_P_ possesses an incomplete immunoglobulin-like fold that is completed by insertion of an N-terminal donor strand of FimG, the subsequent subunit in pilus assembly^11^. The two-domain architecture of FimH is a prerequisite for catch-bond formation because the interactions between FimH_L_ and FimH_P_ determine the conformational state and ligand-binding properties of FimH_L_ (refs 12, 16, 17). A ‘compressed' FimH_L_ conformation was observed in the crystal structure of FimH in the context of the type 1 pilus tip fibrillum in the absence of ligands, with an open binding site and interactions to FimH_P_ mediated via three loop segments: the swing (amino acids (aa.) 27–33), linker (aa. 154–160) and insertion loops (aa. 112–118)^17^. In contrast, an ‘extended' FimH_L_ conformation was observed in crystal structures of the isolated, ligand-bound FimH_L_ domain^18^,^19^,^20^,^21^,^22^,^23^ and in the complex between FimH and the pilus assembly chaperone FimC, where FimC prevents the interactions between FimH_L_ and FimH_P_ (ref. 24). This extended form of FimH_L_ is characterized by a closed ligand-binding pocket and rearranged swing, linker and insertion loops.

Notably, isolated FimH_L_ was reported to show a ligand-binding affinity about two orders of magnitude higher than that of full-length FimH in the tip fibrillum^17^,^25^. Together with mutagenesis experiments disrupting the interdomain interface^26^, these data indicated that ligand-binding is linked to domain separation in FimH, and that mechanical force shifts the ligand-binding affinity towards that of the isolated FimH_L_. However, fundamental aspects of the mechanism underlying the force-dependent binding of FimH remained unknown: (i) How is domain-associated, full-length FimH interacting with ligands? (ii) Does ligand-binding directly induce domain separation? (iii) How are interdomain interactions linked to the ligand-binding affinity of FimH and the kinetics of ligand-binding and dissociation?

To address these questions, we designed a stable, soluble variant of full-length FimH that is equivalent in its structural and functional properties to those of FimH in the assembled fimbrial tip. This variant allowed us to obtain high-resolution structural snapshots of all functional states of FimH and to obtain a complete characterization of ligand-binding kinetics in solution. Together with molecular dynamics simulations, these data reveal a three-state mechanism of FimH catch-bond formation. FimH_P_ accelerates ligand release from FimH_L_ via dynamic allostery by 100,000-fold. In addition, using single-cell tracking experiments, we show that the modulation of ligand affinity by FimH_P_ is not only required for adhesion under mechanical stress, but also for efficient bacterial surface motility in the absence of shear force. Our results provide a first complete structural and kinetic description of a catch-bond system and establish a framework for the analysis of the distinct catch-bond mechanisms in other systems, which also commonly couple interdomain interactions to ligand affinity.

## Results

### Construction of a peptide-complemented FimH

**Figure 1:**
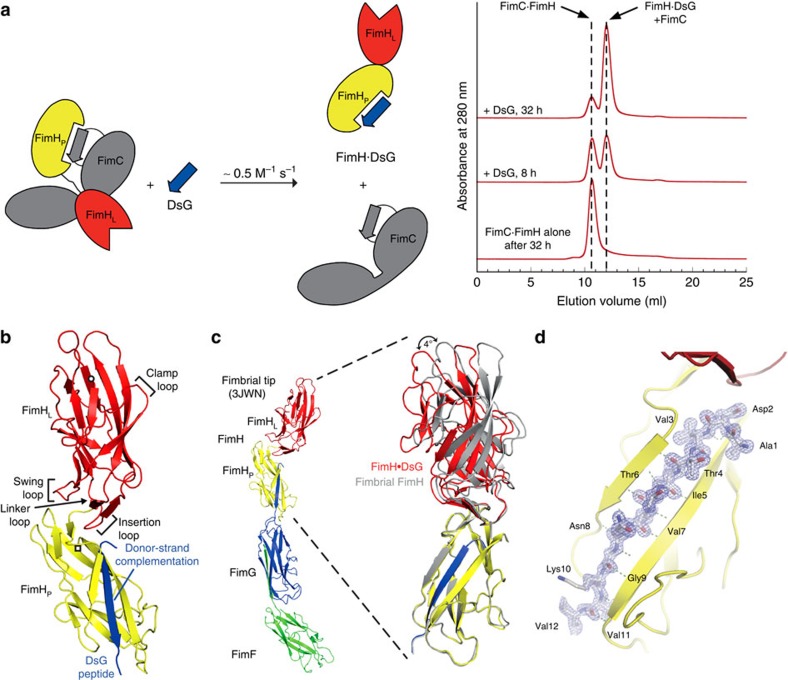
FimH·DsG resembles fimbrial tip FimH. (**a**) Preparation of the FimH·DsG complex by DSE. Left: reaction scheme of the DSE reaction, in which DsG displaces the FimC chaperone from the FimH pilin domain. Right: kinetics of the FimH·DsG complex formation at 37 °C, monitored by analytical gel filtration. DSE was initiated by mixing the FimC·FimH complex (15 μM) with excess DsG peptide (50 μM). Samples were removed after different incubation times, rapidly cooled on ice and immediately subjected to gel filtration. The reaction can be followed by the decrease in the FimC·FimH complex concentration and the simultaneous increase in the concentrations of FimH·DsG and free FimC (FimC and FimH·DsG coelute as a single peak at ∼12 ml). The chromatogram at the bottom shows that the FimC·FimH complex is stable against dissociation/aggregation under the chosen conditions. The rate constant of DSE estimated from these data is ∼0.5 M^−1^ s^−1^. (**b**) Structure of FimH^F18^·DsG (lectin domain FimH_L_, red; pilin domain FimH_P_, yellow; DsG, blue; circle and square indicate N- and C termini, respectively). (**c**) FimH from the fimbrial tip structure (left, PDB ID: 3JWN (ref. 17); FimG, blue; FimF, green) is superposed onto FimH^F18^·DsG based on their pilin domains (aa. 160–279), in the superposition (right) fimbrial FimH is shown in grey. (**d**) Close-up on the DsG peptide (stick representation) bound to FimH^F18^·DsG with 2*F*_o_–*F*_c_ electron density map. Backbone hydrogen bonds of the DsG peptide and β-strands 2 (β2) and 9 (β9) of FimH_P_ are indicated.

Isolated FimH with its non-complemented pilin domain is only marginally stable and shows aggregation tendency under physiological conditions^27^. To establish a stable, isolated FimH molecule with all properties of FimH in the tip fibrillum, we complemented FimH_P_ with the donor-strand peptide of FimG (FimG residues 1−14; termed DsG). The FimH·DsG complex was obtained in good yields and purified after an *in vitro* reaction, mimicking the first donor-strand exchange (DSE) reaction during pilus assembly *in vivo*. In this reaction, the FimG donor strand displaces the pilus assembly chaperone FimC from FimH (Fig. 1a):


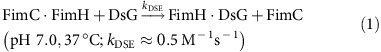


The experiments described in the following were performed with FimH from the faecal *E. coli* strain F18 (FimH^F18^), which is structurally identical to the most prevalent variants in uropathogenic infection^25^, and FimH from the wild-type *E. coli* strain K12 (FimH^K12^), which differ in three amino acids in FimH_L_ (K12→F18: Val27Ala, Asn70Ser, Ser78Asn; Supplementary Fig. 1a). The isolated lectin domains (residues 1–159) of both FimH variants (FimH_L_^K12^ and FimH_L_^F18^) were produced by direct expression in the *E. coli* periplasm and were purified as described^27^.

### Ligand-free FimH·DsG resembles FimH in the fimbrial tip

The crystal structure of the binary complex FimH^F18^·DsG was determined at atomic resolution by molecular replacement (Table 1). FimH^F18^·DsG comprises the jellyroll fold FimH_L_ and the immunoglobulin-like FimH_P_ domain complemented with the FimG donor strand (Fig. 1b and Supplementary Fig. 1b). It closely resembles unliganded FimH in the fimbrial tip complex (Fig. 1c)^17^, with a root-mean-square deviation of C_α_ positions (C_α_ r.m.s.d.) of 1.1 Å. The individual FimH_P_ and FimH_L_ domains are even more closely resembling unliganded, fimbrial FimH (r.m.s.d. 0.45 and 0.55 Å, respectively) and undergo only a minimal hinge-bending rotation of 4° (Fig. 1c).

The DsG peptide in FimH^F18^·DsG is in identical position as compared with the N-terminal FimG extension in the fimbrial tip structure; it interacts with β-strand 2 and 9 of FimH_P_ (Fig. 1d). All contacts in the FimH_L_–FimH_P_ interdomain region (Supplementary Fig. 1c,d)^17^ as well as the conformation of the empty ligand-binding pocket observed in FimH in the fimbrial tip are preserved in FimH^F18^·DsG. Thus, FimH^F18^·DsG represents the ligand*-***free** state of fimbrial FimH, with **A**ssociated FimH_L_ and FimH_P_ (**A**_**free**_ state) and is an elegant minimal system to analyse the crosstalk between ligand-binding and interdomain interactions underlying the formation of catch-bonds by FimH.

### Persistence of domain association in ligand-bound FimH·DsG

**Figure 2:**
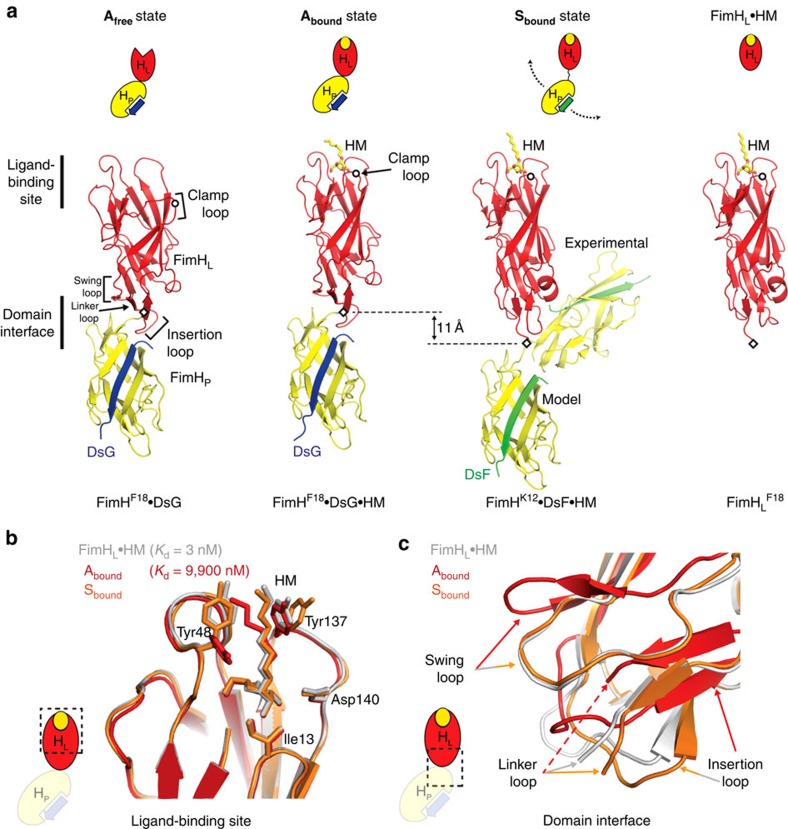
Crystallographic analysis of FimH conformational states. (**a**) FimH^F18^·DsG in the **A**_**free**_ (left) and in the **A**_**bound**_ states (FimH^F18^·DsG·HM) in comparison with the **S**_**bound**_ state of FimH^K12^·DsF·HM and the isolated FimH_L_^F18^·HM (right). The FimH_L_, FimH_P_, DsF and DsG are coloured in red, yellow, green and blue. The experimentally *in crystallo* trapped orientation of FimH_P_ in FimH^K12^·DsF·HM and a modelled position based on a hinge motion stretching around Gly157 is indicated. A schematic representation for each crystal structure, similar to Fig. 1a, is given. The tip of the clamp loop and the C terminus of FimH_L_ are indicated as a circle and diamond, respectively. (**b**) Comparison of the conformation of the ligand-binding site in the **A**_**bound**_ (red) and **S**_**bound**_ (orange) states with the isolated lectin domain FimH_L_ (grey) and (**c**) comparison of the interdomain interface of the lectin domain.

To test whether ligand-binding causes domain separation in FimH, we determined the co-crystal structure of the ternary complex of FimH^F18^·DsG with *n*-heptyl α-D-mannoside (HM), an established model ligand of FimH^20^, as well as crystal structures of the isolated FimH_L_^F18^ and FimH_L_^K12^ lectin domains in complex with HM (Table 1). FimH^F18^·DsG·HM adopts the same closed conformation of the ligand-binding site as previously observed in other FimH_L_–ligand complexes (Fig. 2a,b and Supplementary Fig. 2)^28^. The mannopyranose moiety of HM is coordinated by the side chains of Asp54, Gln133, Asn135 and Asp140, and the main chain of Phe1 and Asp47, and the *n*-heptyl aglycone of HM is sandwiched between Tyr48 and Tyr137. Compared with the **A**_**free**_ form, all loops surrounding the binding pocket close down onto the HM ligand. The most substantial conformational difference to **A**_**free**_ is observed for the clamp loop (aa. 8–16), whose tip moves almost 6 Å towards HM (Supplementary Fig. 1e,f).

Besides the closing of the ligand-binding pocket, the overall conformation of ligand-free FimH_L_ in **A**_**free**_ and HM-bound FimH^F18^·DsG is closely similar (C_α_ r.m.s.d. 1.1 Å; Fig. 2a,b and Supplementary Fig. 2). Unexpectedly, the structural change in the ligand-binding site in FimH^F18^·DsG·HM was not transmitted to the domain interface, where the interdomain contacts and the conformations of the swing, linker and insertion loops remained intact. The lectin domain in the FimH^F18^·DsG·HM complex thus differs drastically from HM-bound isolated FimH_L_ domains with respect to the swing, linker and insertion loop conformations (Fig. 2c). On the basis of the persistence of the domain **A**ssociation in the ligand-**bound** form, this state of FimH was termed **A**_**bound**_.

To test the stability of the **A**_**bound**_ state against domain separation and to exclude potential effects of selective crystallization, a molecular dynamics (MD) simulation of the **A**_**bound**_ state was conducted using the CHARMM36 force field of the NAMD package (Supplementary Fig. 3a,b). The domain association remained intact over 100 ns of simulation time without substantial changes in the domain interface; fluctuations were limited to the clamp loop region close to the ligand-binding site. On *in silico* removal of the HM ligand after initial equilibration, the **A**_**bound**_ state underwent a spontaneous transition to the **A**_**free**_ state after ∼75 ns of simulation time via an opening of the clamp loop (Supplementary Fig. 3c,d), reproducing the experimentally observed dependence of the binding-site conformation on ligand-binding. Thus, the MD simulations indicate that **A**_**bound**_ is a stable conformational state of FimH induced by ligand-binding.

### Trapping of a domain-separated state of full-length FimH

The increase in apparent affinity of FimH to its target glycans under tensile mechanical forces^12^,^29^ has previously been linked to a separation of the FimH_L_ and FimH_P_ (ref. 17). To trap a potential domain-separated state of FimH for structural characterization in the absence of tensile force, we considered FimH variants with weakened interdomain interactions. We had shown previously that FimH_P_ also accepts the donor strand of the non-cognate subunit FimF (DsF). However, FimH_P_ is slightly less stabilized by complementation with DsF than with the natural donor-strand DsG^30^. We hypothesized that such complementation with DsF instead of DsG could also result in a mild destabilization of the interdomain interface in full-length FimH.

**Figure 3:**
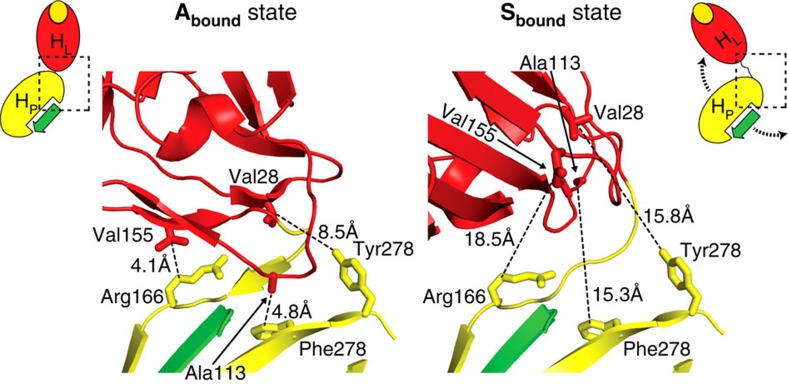
The interdomain region in the S_bound_ state. Close-up of the interdomain region of FimH^K12^·DsF·HM in the **A**_**bound**_ form (left) and FimH^K12^·DsF·HM in the **S**_**bound**_ state (right). A cartoon representation for each crystal structure, similar to Fig. 1a, is given. Key residues in the interface are shown as sticks. FimH_P_, FimH_L_ and DsF are coloured in yellow, red and green, respectively.

We then determined the co-crystal structure of FimH^K12^·DsF with HM (FimH^K12^·DsF·HM) at 3.0 Å resolution with four molecules in the asymmetric unit. Three FimH^K12^·DsF·HM molecules closely resembled the **A**_**bound**_ state (r.m.s.d. of 0.6 Å to FimH^F18^·DsG·HM) with a preserved interdomain interface. In the fourth molecule, however, the FimH_L_ and FimH_P_ domains were separated and they adopted a drastically different relative orientation with an angle between the domains of ∼45° instead of ∼150° in the other three molecules (Fig. 2a). FimH_P_ is virtually identical in all four FimH molecules in the crystal (r.m.s.d. 0.4 Å). In contrast, the FimH_L_ domain differs significantly between the fourth, domain-separated and the three full-length FimH molecules in the crystal. It shows closest similarity to the isolated FimH_L_·HM (r.m.s.d. 0.45 Å); in particular, all interdomain loops adopt identical conformations, which are incompatible with domain association (Figs 2c and 3). Remarkably, in the bent fourth molecule, no interactions between FimH_L_ and FimH_P_ other than the direct covalent linkage are detected, equivalent to a breakdown of the total 500 Å^2^ interdomain interface of the **A**_**bound**_ state (Fig. 3). This molecule thus represents a third state, the domain-**S**eparated, ligand-**bound** state of FimH, **S**_**bound**_. The complete absence of non-covalent interdomain interactions indicates that the **S**_**bound**_ state does not possess a defined relative domain orientation in solution, and that the observed, kinked conformation has been selected only by crystal packing.

To analyse the transition trajectory of the **A**_**bound**_ to the **S**_**bound**_ state, we removed the FimH_P_ domain after equilibration from the **A**_**bound**_ state *in silico* for a 180-ns molecular dynamics simulation (Supplementary Fig. 3e,f). In contrast to the transition between the **A**_**bound**_ and **A**_**free**_ states on ligand removal, a sharp transition to the conformation of FimH_L_ in the **S**_**bound**_ state was not observed. The conformation only slowly moved towards **S**_**bound**_; however, the FimH_L_ loops that had formed in the former interdomain interaction kept fluctuating throughout the simulation, indicating lower cooperativity and potentially a higher activation energy for the **A**_**bound**_→**S**_**bound**_ compared with the **A**_**bound**_→**A**_**free**_ transition.

A comparison of the structural dynamics in the **A**_**bound**_ and **S**_**bound**_ states clearly reveals differences in the FimH_L_–FimH_P_ interface region. The root-mean-square fluctuations of atom positions (r.m.s.f.) increase in the swing and insertion loop from a background level of ∼0.7 Å in **A**_**bound**_ to 1.5 and 2 Å in **S**_**bound**_, respectively. Surprisingly, despite the virtually identical conformations of the entire ligand-binding site depicted by X-ray crystallography (Fig. 2b), the clamp loop, which exhibits the most significant conformational changes between the open and closed conformations, exhibits strongly reduced fluctuations in **S**_**bound**_, with r.m.s.f. decreasing by up to 1.5 Å (Supplementary Fig. 3g,h). This change in clamp loop dynamics provides a mechanistic link between domain association and ligand-binding in full-length FimH.

### Domain association alters FimH–ligand-binding kinetics

**Figure 4:**
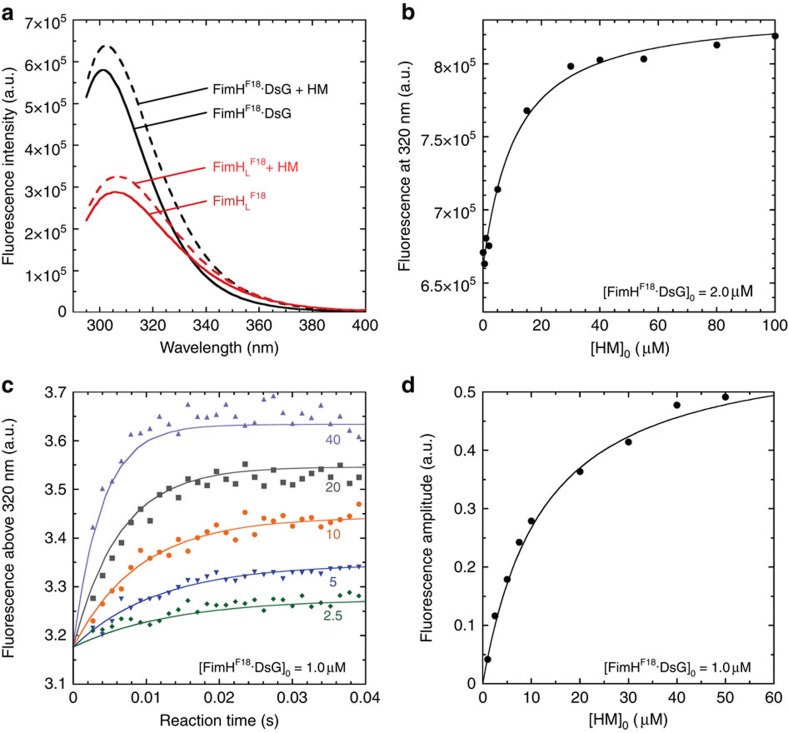
Kinetics of HM binding and release by full-length FimH. (**a**) Fluorescence spectra (excitation at 280 nm) of FimH_L_^F18^ (2 μM; red lines) and FimH^F18^·DsG (2 μM; black lines) in the absence (solid lines) or presence of 200 μM HM (dotted lines). (**b**) Equilibrium titration of FimH^F18^·DsG (2 μM) with HM, recorded via the fluorescence increase at 320 nm. The total concentration of HM is plotted against the recorded fluorescence signal. Data were fitted (solid line) according to equation (2) (*cf.* experimental section) and yielded a *K*_d_ value of 9.9±1.5 μM. (**c**) Stopped-flow fluorescence kinetics of HM binding to FimH^F18^·DsG (1.0 μM), recorded via the fluorescence change above 320 nm. The HM concentration was varied between 0 and 50 μM. Five representative traces are shown (HM concentrations are given in μM). The fluorescence traces were globally fitted according to a second-order binding and first-order dissociation reaction (solid lines; Table 2). (**d**) Amplitudes of the reactions monitored in **c**, plotted against the total HM concentration. Data were fitted (solid line) according to equation (2), yielding a *K*_d_ value of 12±1 μM.

To analyse the ligand-binding properties of FimH·DsG, we exploited the increase in intrinsic tryptophan fluorescence in the FimH·DsG complexes of ∼10% on HM binding (Fig. 4a). This difference was used to measure the dissociation constant of HM binding by equilibrium titration (Fig. 4b) and the rates of HM binding and dissociation by stopped-flow fluorescence kinetics (Fig. 4c,d). The FimH·DsG constructs showed uniform binding and dissociation kinetics, consistent with the view that domain-separated states of FimH are not significantly populated in the absence of shear force. The results revealed equilibrium dissociation constants (*K*_d_) of 3.6 and 9.9 μM for FimH^K12^·DsG and FimH^F18^·DsG, respectively (Table 2). HM binding to FimH·DsG is extremely dynamic and was characterized by fast association rates (*k*_on_) of 5.0 × 10^6^ and 4.9 × 10^6 ^M^−1^s^−1^, respectively, and rapid dissociation reactions (Supplementary Fig. 4). The rates of HM dissociation (*k*_off_) of 22 and 58 s^−1^ for FimH^K12^·DsG and FimH^F18^·DsG translate into dissociation half-lives of only 32 and 12 ms, respectively.

**Figure 5:**
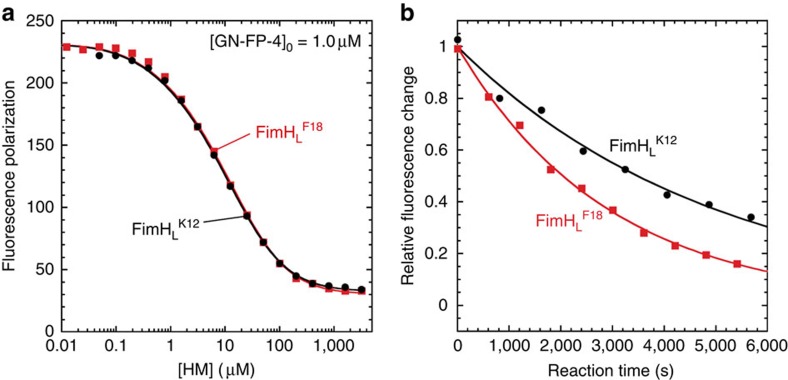
HM binding and release by the isolated FimH lectin domain FimH_L_. Analysis of FimH_L_·HM interactions based on competition between HM and the synthetic fluorescent GN-FP-4 ligand. (**a**) HM binding to FimH_L_ analysed by displacement of GN-FP-4 from FimH_L_ variants as indicated. An equimolar mixture of FimH_L_ and GN-FP-4 (1 μM each) was incubated with different HM concentrations (10 nM–3.2 mM) for >18 h. GN-FP-4 displacement is monitored by a decrease in fluorescence polarization at 528±20 nm (excitation at 485 nm). Data were fitted (solid lines) according to a mechanism in which two ligands compete for the same binding site, with fixed *K*_d_ values for GN-FP-4 binding (*cf.* Table 2). (**b**) Kinetics of HM dissociation from FimH_L_. A solution with equimolar concentrations of FimH_L_ and HM (3 μM each, guaranteeing >95% occupancy with HM) was mixed with excess GN-FP-4 (10 μM), and the decrease in GN-FP-4 fluorescence at 520 nm as a consequence of HM dissociation and GN-FP-4 binding was recorded (Supplementary Fig. 5f–j). The obtained first-order kinetics are independent of the GN-FP-4 concentration and thus directly monitor HM dissociation.

In contrast to full-length FimH, isolated FimH_L_^K12^ showed no change in tryptophan fluorescence on HM binding. We therefore determined the HM affinity of isolated FimH_L_ indirectly by a competition experiment based on a newly designed fluorescent ligand, the fluorescein-labelled α-D-mannoside GN-FP-4 (Supplementary Fig. 5a–e and Supplementary Note 1). Displacement of GN-FP-4 from FimH_L_ by increasing HM concentrations under equilibrium conditions showed that both FimH_L_^K12^ and FimH_L_^F18^ bind HM with 3,300-fold higher affinity compared with the respective FimH·DsG complexes (*K*_d_ values 1.1 and 3.0 nM, respectively; Fig. 5a and Table 2). In an inverse competition experiment (Supplementary Fig. 5f–j), in which HM in preformed FimH_L_·HM complexes was displaced by GN-FP-4, off-rates of 2.0 × 10^−4^ and 3.5 × 10^−4 ^s^−1^ were determined for FimH_L_^K12^ and FimH_L_^F18^, respectively, corresponding to dissociation half-lives of 58 and 33 min (Table 2). On the basis of these measured off-rates and equilibrium dissociation constants, *k*_on_ rates of 1.8 × 10^5^ and 1.2 × 10^5 ^M^−1 ^s^−1^ were calculated for FimH_L_^K12^ and FimH_L_^F18^, respectively. The on-rates for the isolated FimH_L_ domains are thus 30-fold lower than those of the corresponding full-length FimH·DsG complexes.

Together, these results demonstrate that the 3,300-fold higher affinity of the isolated FimH_L_ compared with full-length FimH results from a more than 100,000-fold lower ligand dissociation rate in isolated FimH_L_, combined with a ligand-binding rate reduced by only 30-fold (Table 3). The 3,300-fold higher affinity for HM of FimH_L_ relative to FimH·DsG translates into a free energy of 20 kJ mol^−1^ for the interaction between FimH_L_ and FimH_P_ in full-length FimH. This corresponds very well with the mechanical work required for domain separation, as a displacement of FimH_L_ from FimH_P_ by 11 Å for complete domain separation (Fig. 2a)^17^, and a force of 40 pN required to populate the domain-separated state of FimH^31^ yields a value of 26.5 kJ mol^−1^.

### Domain association in FimH promotes bacterial motility

**Figure 6:**
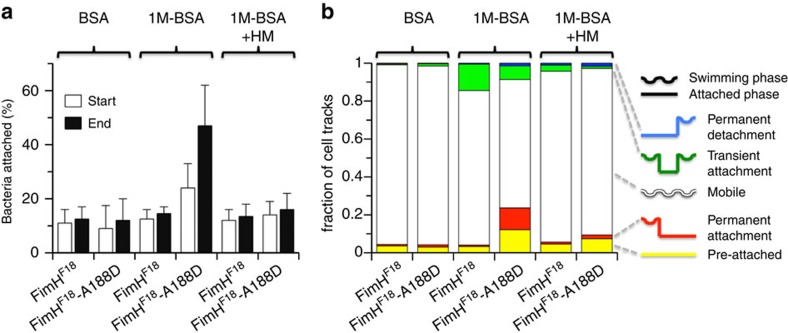
Cell-tracking analysis of bacterial motility on mannosylated surfaces. *E. coli* cells piliated with FimH^F18^ or the FimH^F18^-Ala188Asp variants were tracked under static conditions in the absence of shear force. (**a**) The fraction of bacteria attached to mannose-coated (1M-BSA) or BSA-coated surface (negative control) at the beginning of the time-lapse movies (white bars) and after 5 min (black bars) are given. Bacterial motility on 1M-BSA was analysed in the absence and presence of HM. The delay between application of bacteria and movie recording was ∼1 min. (**b**) Fraction of tracked cells that were pre-attached (yellow; speed <0.5 μm s^−1^), permanently attach (red), were mobile (white), transiently attach (green) or permanently detach (blue) during the entire observation time (5 min). Right: schematic depiction of the observed cell behaviour. FimH^F18^-piliated *E. coli* show almost exclusively transient attachment events on 1M-BSA. FimH^F18^-Ala188Asp-piliated *E. coli* show less transient attachment but enhanced permanent attachment to 1M-BSA. Transient and permanent attachment to 1M-BSA is significantly reduced in the presence of HM. For each experiment five to seven independent replicates were analysed.

Uropathogenic *E. coli* require firm adhesion to the urinary epithelium under flow conditions to escape clearance by urine excretion. On the other hand, bacterial adhesion must be weak enough in the absence of external shear to allow flagellar motility as a prerequisite for the invasion of new tissue areas^32^,^33^. While the role of FimH catch-bond binding for adhesion under flow conditions had clearly been demonstrated^12^,^29^,^34^, the relevance of rapid ligand dissociation under static conditions for flagellar motility remained unclear because of the complex interplay of flagellar swimming and the avidity of multivalent surface interactions by hundreds of *E. coli* pili. Here we employed single-cell tracking of piliated *E. coli* cells moving on surfaces coated with mono-mannosylated bovine serum albumin (1M-BSA), an established model system for analysing FimH-based adhesion^12^,^29^, for a classification of cell motility into two states, attached or mobile (for details see Methods and Supplementary Fig. 6). To study the influence of FimH interdomain interactions, we compared isogenic *E. coli* strains producing either wild-type FimH^F18^ or the FimH^F18^-variant Ala188Asp, which is characterized by a destabilized interaction between FimH_P_ and FimH_L_ (ref. 26) and serves here to mimic the **S**_**bound**_ state in the absence of shear force^35^. The overall fraction of adherent FimH^F18^-piliated bacteria on 1M-BSA-coated surfaces was identical to background levels on non-adhesive BSA-coated surfaces at 10–12% of tracked bacteria (Fig. 6a). In contrast, FimH^F18^–Ala188Asp-piliated bacteria showed an increased fraction of adherent cells of 24% already at the beginning of cell tracking after a 1-min dead time, which further increased during the 5-min observation period to 48% (Fig. 6a and Supplementary Movies 1 and 2).

Cell tracking permitted quantitative analysis of the transition of individual cells between a mobile and an attached state (Supplementary Fig. 7). On non-adhesive control surfaces coated only with BSA, less than 2% of all FimH^F18^- or FimH^F18^–Ala188Asp bacteria showed shifts between the two states of motion (Fig. 6b and Supplementary Fig. 7c). However, on adhesive 1M-BSA surfaces, 13.9% of all FimH^F18^ tracks (green in Fig. 6b) exhibited a single transient attachment event with a mean duration of 6.9 s (Supplementary Fig. 7d). For FimH^F18^–Ala188Asp-piliated bacteria, only 7.2% of the cells showed attachment/detachment, but with fivefold longer adhesion (35.2 s; Supplementary Fig. 7d). Remarkably, the fraction of cells that permanently stayed attached after adhesion to 1M-BSA until the end of the observation period was much larger for FimH^F18^–Ala188Asp (11.5%) than for FimH^F18^ (0.6%; red in Fig. 6b). Those permanently attached cells escape kinetic analysis; thus, the true average attachment time for FimH^F18^–Ala188Asp must be considerably larger than 35.2 s. Permanent attachment is also the main cause of the increased fraction of attached cells for FimH^F18^–Ala188Asp-piliated bacteria (Fig. 6a).

Altogether, cell-tracking analysis revealed that enforced domain separation in the FimH^F18^–Ala188Asp variant resulted in reduced detachment rates and a larger proportion of permanently attached cells. These results directly demonstrate, at the cellular level, the importance of fast, spontaneous ligand dissociation catalysed by interdomain allostery in FimH–ligand complexes for bacterial motility in the absence of tensile mechanical forces.

## Discussion

**Figure 7:**
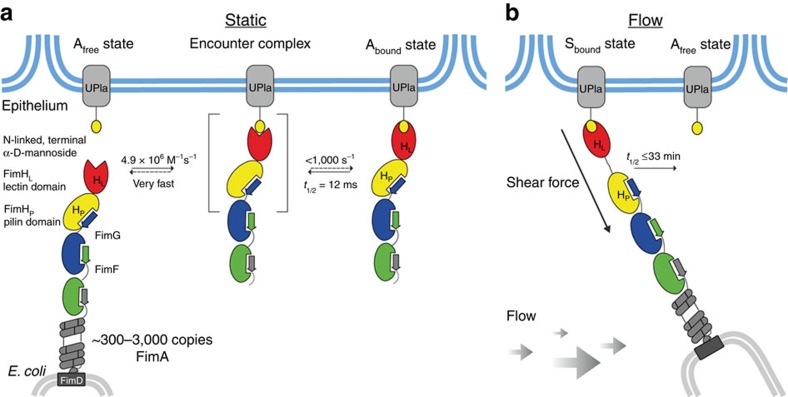
Catch-bond mechanism of FimH-mediated cell adhesion. (**a**) In the absence of tensile mechanical force, formation of the FimH-Uroplakin 1a (UPIa) complex comprises the highly dynamic transition of the **A**_**free**_ to the **A**_**bound**_ state. The reaction likely proceeds via a transient encounter complex (indicated in square brackets). The reaction of the encounter complex to **A**_**bound**_ is not rate-limiting and must have a half-life of less than 1 ms. Dissociation of the receptor from the FimH lectin domain in the **A**_**bound**_ state is promoted via dynamic allostery by the pilin domain that acts as a negative allosteric regulator. The reaction from **A**_**bound**_ to the encounter complex corresponds to *k*_off_. Fast binding and release of UPIa by FimH enables bacterial motility on the bladder epithelium. (**b**) Shear force increases the population of the **S**_**bound**_ state of FimH, in which the pilin and lectin domains are separated. The dissociation of **S**_**bound**_ under shear force is slowed down up to 100,000-fold compared with **A**_**bound**_. The indicated rate constants and half-lives correspond to the interaction between FimH^F18^ and the model ligand HM. Rate limiting reactions are indicated by solid arrows, and fast, non-limiting reactions by dashed arrows.

The characterization of full-length FimH had so far been restricted to the analysis of the adhesive properties of piliated *E. coli* cells and binding studies with the purified type 1 pilus tip fibrillum. With the FimH·DsG complex, we have now established a model system for quantitative studies of the interaction of FimH with carbohydrate ligands. Soluble FimH·DsG efficiently mimicks FimH in the context of the assembled tip fibrillum, is readily available in milligram quantities and permits the determination of ligand-binding and release kinetics in solution. Using FimH·DsG, we obtained high-resolution snapshots of three functionally relevant states of FimH (Fig. 7). In the absence of ligands, FimH adopts the **A**_**free**_ state with associated FimH_L_ and FimH_P_ and an open conformation of the ligand-binding site, which is responsible for the 30-fold faster ligand-binding of full-length FimH as compared with the isolated FimH_L_ domain. Ligand-binding in the absence of shear force induces the **A**_**bound**_ state with a closed binding site. In contrast to earlier hypotheses^17^, the transition from **A**_**free**_ to **A**_**bound**_ is restricted to the ligand-binding site, while all interactions between FimH_L_ and FimH_P_ observed in the **A**_**free**_ state remain preserved in **A**_**bound**_. The **A**_**free**_→**A**_**bound**_ transition most likely follows an induced fit mechanism, in which the formation of an encounter complex between FimH·DsG and HM is rate-limiting and followed by a fast, unimolecular rearrangement to the **A**_**bound**_ state, in agreement with the observation that binding of the model ligand HM remained rate-limiting for the formation of **A**_**bound**_ even at the highest HM concentrations used. Stopped-flow-binding kinetics indicate that the lifetime of the proposed encounter complex before **A**_**bound**_ formation is below 1 ms (Fig. 4c). Under tensile mechanical force applied to the FimH–ligand complex, mimicked here by the destabilized variant FimH·DsF and crystal packing forces, the domain-separated state of FimH, **S**_**bound**_, is formed. In the **S**_**bound**_ state, FimH_L_ and FimH_P_ no longer interact specifically and are only connected via the linker segment comprising FimH residues 154–160. In this **S**_**bound**_ state, FimH_L_ adopts a conformation closely resembling isolated FimH_L_ with bound ligand.

Notably, ligand dissociation from FimH·DsG is 100,000-fold faster than that from the isolated FimH_L_ domain. This is striking because the respective crystal structures revealed indistinguishable ligand interactions and binding-site conformations in the FimH·DsG·HM and FimH_L_·HM complexes (Fig. 2b). MD simulations identified a considerable increase in the conformational dynamics of the FimH_L_–ligand-enclosing clamp loop in the **A**_**bound**_ state as the most likely cause of the dramatic increase in *k*_off_ in the FimH·DsG·HM complex. The altered dynamics in FimH_L_ in the **A**_**bound**_ state are the result of the presence of FimH_P_, which can be described as a negative allosteric regulator^36^,^37^,^38^. The allosteric communication from the FimH_P_–FimH_L_ interface to the ligand-binding site reaches over 40 Å, and is mediated via changes in protein dynamics rather than in static structure, in line with a general model of dynamic allostery^39^,^40^. Our data demonstrate that the interdomain interactions in FimH (i) maintain the open conformation of the binding pocket and guarantee rapid ligand-binding and (ii) intramolecularly catalyse ligand dissociation by more than 100,000-fold. Rapid ligand-binding and short lifetimes of the FimH ligand complex allow for rapid dissociation of individual pili from their ligands in the absence of shear force. Our biophysical data demonstrate that this mechanism is conserved between the K12 and the F18 *E. coli* strains.

Different mechanistic models, such as the two-pathway^41^, the deformation^42^ and the sliding re-binding model^43^, have been developed to describe catch-bond interactions, often based on powerful single-molecule atomic force measurements. These models included the principle of allosteric control of ligand-binding affinity^26^,^31^, which was clearly fully confirmed in our present study. However, these conceptual models did not reveal the underlying atomic-scale mechanisms in different catch-bond systems. For most catch-bond systems, including the cadherin–catenin binding to actin filaments^3^,^44^, integrin epithelial cell adhesion^45^,^46^ and TCR–MHC interactions^6^,^7^,^47^, structural information is, if at all, available only for one state or from computer simulations. One exception is the selectins, which employ catch-bond binding for leukocyte recruitment. Selectins are multidomain cell surface receptors, which consist of a lectin domain for complex carbohydrate binding, linked via an epidermal growth factor (EGF)-like domain to a variable number of short consensus repeat domains and a transmembrane-anchoring helix. Selectins exist in two conformations, a bent and an extended one, which differ in the angle between their lectin and EGF-like domain. Ligand-binding and conformational changes in the ligand-binding site are directly linked via a complex allosteric coupling mechanism to the adoption of the extended conformation^48^,^49^. Tensile mechanical force under flow conditions acts along the axis of the ligand-binding site and the Lec-EGF interface resulting in a stabilization of the extended conformation and thus increased ligand complex lifetimes^2^,^49^. Moreover, in FimH, catch-bond behaviour is mediated by the interplay of a lectin and an anchoring domain that does not interact with the ligand. Ligand-binding by FimH in the absence of shear force results in a closing of the ligand-binding site, but, in contrast to selectins, is not directly linked to altered interdomain interactions. Here mechanical force promotes domain separation and completely releases FimH_L_ from FimH_P_, which acts as an activator of ligand release via dynamic allostery. Remarkably, selectins and the fimbrial adhesin FimH thus employ entirely different mechanisms for establishing catch-bond behaviour by crosstalk between a lectin and an anchoring domain that provides tethering to a shaft. In both systems, the selectins and fimbrial adhesion, the shaft structures linking the terminal lectin/coupling domains to the cell surface, may contribute to the overall catch-bond behaviour, either via directly influencing coupling domain behaviour or via their general elastic properties^50^,^51^.

The cell-tracking experiments indicate the importance of rapid ligand release from the high-mannose-type glycoprotein receptor uroplakin 1a in the lower urinary tract^52^ for flagellar motility of piliated bacteria, and hence their ability to colonize new tissue areas under certain conditions during infection^12^,^29^,^53^. This provides a plausible explanation for the fact that low-affinity FimH variants were preserved in numerous uropathogenic *E. coli* strains. Binding of terminal mannoses with low affinity in the absence of shear force may also play a role in preventing the clearance of uropathogenic *E. coli* from the urinary tract by competitive binding to the Tamm–Horsfall protein in the urine^54^. In turn, populating the **S**_**bound**_ state with an extremely low dissociation rate ensures tight bacterial adhesion under the mechanical forces of urine excretion. FimH is a promising target for anti-adhesive therapy of UTI because FimH antagonists, in contrast to antibiotics, are not exerting selection pressure towards resistance formation^18^,^55^,^56^. Previous ligand-binding studies on the isolated FimH_L_ domain mimic the domain-separated **S**_**bound**_ state of FimH. This state is characterized by extremely low off-rates and is promoted *in vivo* only after ligand-binding and the onset of flow conditions. Our kinetic data on ligand dissociation from full-length FimH demonstrate that rapid, competitive displacement of FimH from its carbohydrate ligands by FimH antagonists is well possible in the absence of shear force. Thus, full-length FimH (for example, in the form of the FimH·DsG complex established in this study) instead of the isolated FimH_L_ domain is the relevant target for the development of anti-adhesive drugs. Importantly, the concept of the FimH·DsG model system can now be expanded to other related adhesive pilus adhesins. In combination with the novel fluorescent GN-FP-4 ligand, this model system paves the way for efficient screening for anti-adhesive drug candidates.

## Methods

### Materials

The synthetic DsG (sequence: ADVTITVNGKVVAKR) and DsF peptide (sequence: ADSTITIRGYVRDNG; >95% purity) were purchased from JPT (Germany). Guanidinium chloride (‘AA-Grade' for spectroscopy) was obtained from NIGU Chemie (Germany). Standard chemical of highest purity available was obtained Sigma, Merck or AppliChem. If not mentioned otherwise, chromatography media for protein purification were purchased from GE Healthcare (UK). Oligonucleotides were from Microsynth (Switzerland).

### Construction of expression plasmids

Expression plasmids for the periplasmic production of the *E. coli* F18 FimH lectin domain (FimH_L_^F18^) and for the periplasmic co-expression of full-length FimH^F18^ with FimC were based on the expression plasmids pfimH_L_ and pfimH-fimC-ATG, respectively, for the analogous proteins from *E. coli* K12 (ref. 27). Six silent mutations replacing rare codons were introduced into the *E. coli* F18 *fimH* gene (*fimH*^*F18*^) contained in the plasmid pGB2-24 (ref. 57) with the QuikChange mutagenesis kit (Agilent Technologies, Switzerland) to improve periplasmic expression. The coding sequence of the modified *fimH*^*F18*^ gene was amplified by PCR using the primers 5′-GATCCTCTAGAGGAGGGATGATTGTAATGAAACGAG-3′ and 5′-TTTCAAGCTTATTGATAAACAAAAGTCACG-3′ and cloned into pfimH-fimC-ATG^27^ via the XbaI and HindIII sites (thereby replacing the *fimH*^*K12*^ gene) and yielded pfimH^F18^–fimC–ATG. The gene encoding FimH_L_^F18^ was amplified with the primers 5′-GATCCTCTAGAGGAGGGATGATTGTAATGAAACGAG-3′ and 5′-CAGCCAAGCTTAGCCAGTAGGCACCACCAC-3′ and ligated via the XbaI and HindIII sites into ptrc99a-f1-stopp^27^. Protein production in the resulting plasmid pfimH_L_^F18^ is under control of the trc–promotor/*lac* operator.

### Protein production and purification

For purification of the complexes FimC·FimH^K12^ and FimC·FimH^F18^, *E. coli* HM125 harbouring the corresponding co-expression plasmid was grown at 30 °C in 2YT medium containing ampicillin (100 μg ml^−1^). At an OD_600_ of 1.5, isopropyl-β-D-thiogalactoside (IPTG) was added to a final concentration of 1 mM. The cells were further grown for 12−18 h, harvested by centrifugation, suspended in cold 50 mM Tris-HCl pH 7.5, 150 mM NaCl, 5 mM EDTA, 1 mg ml^−1^ polymyxin B sulfate (18 ml l^−1^ of culture) and stirred at 4 °C for 1.5 h. After centrifugation, the supernatant (periplasmic extract) was dialysed against 20 mM Tris-HCl pH 8.0 and applied to a QA52 (Whatman, Maidstone, UK) column equilibrated with the same buffer. The flow-through containing the respective FimC·FimH complex was dialysed against 20 mM MOPS–NaOH pH 7.0, loaded onto a Resource S column equilibrated with the same buffer and the complexes were eluted with a linear NaCl gradient (0−400 mM). Fractions containing FimC·FimH were pooled and loaded onto a Superdex 75 (HiLoad 26/60) column equilibrated with 20 mM NaH_2_PO_4_–NaOH pH 7.4, 50 mM NaCl. Fractions containing the pure complex were pooled and stored at 4 °C until further use. Typically, 3−5 mg of the purified complex were obtained per litre of bacterial culture.

For expression of the isolated *E. coli* FimH_L_^K12^ and FimH_L_^F18^, *E. coli* HM125 transformed with the respective expression plasmid was grown at 30 °C in M9 medium containing ampicillin (100 μg ml^−1^) to an OD_600_ of 1.0, and expression was induced with 1 μM IPTG. After further growth for 12 h, cells were subjected to periplasmic extraction (see above). The extracts were mixed with 0.11 volumes of 1 M acetic acid–NaOH pH 4.5, dialysed against 10 mM acetic acid–NaOH pH 4.5 and then loaded onto a SP-Sepharose column equilibrated with the same buffer. The flow-through was collected and its pH was adjusted to 8.0 by addition of 1 M Tris-HCl pH 8.2. This solution was then applied to a Q-Sepharose column equilibrated with 20 mM Tris-HCl pH 8.0. The flow-through containing FimH_L_ was loaded onto a Resource S column dialysed against 20 mM formic acid–NaOH pH 4.0. The protein was eluted with a linear NaCl gradient (0−1 M). Fractions containing pure FimH_L_ were pooled, dialysed against water and stored at −20 °C. The identity of the purified proteins was confirmed by electrospray ionization (ESI)–mass spectrometry (FimH_L_^F18^: calculated: 16,934.9 Da; measured: 16,935.0 Da; FimH_L_^K12^: calculated: 16,963.0 Da; measured: 16,962.8 Da). About 11 mg of the pure FimH_L_ was obtained per litre of bacterial culture.

### Production of FimH·DsG and FimH^K12^·DsF complexes

The respective FimC·FimH complex (40 μM) was incubated with a threefold molar excess of the DsG peptide and incubated in 20 mM NaH_2_PO_4_–NaOH, pH 7.0, 50 mM NaCl for 48 h at 37 °C. The reaction mixture containing isolated FimC, the FimH·DsG complex and excess DsG was dialysed against 20 mM acetic acid–NaOH pH 4.5 and loaded onto a Resource S (6 ml) column equilibrated with the same buffer. The FimH·DsG complex was eluted with a linear NaCl gradient (0−400 mM). Fractions containing the pure complex were pooled, dialysed against water and stored at 4 °C. The FimH·DsG partially dissociated during ESI–mass spectrometry analysis, so that masses of the intact complexes and free FimH were obtained: FimH^K12^·DsG: calculated mass: 30,635.3 Da; measured mass: 30,636.0 Da; FimH^F18^·DsG: calculated mass: 30,607.3 Da; measured mass: 30,607.0 Da; FimH^K12^: calculated mass: 29,064.5 Da; measured mass: 29,064.0 Da; FimH^F18^: calculated mass: 29,036.4 Da; measured mass: 29,036.0 Da. The overall yields of the purified FimH·DsG complexes relative to the initial amount of FimC·FimH were in the range of 50–55%. The FimH^K12^·DsF complex was generated and purified as described for the FimH·DsG complexes (FimH^K12^·DsF: calculated mass: 30,702.2 Da; measured mass: 30,702.5 Da). The FimH·DsF complex was prepared from the FimC·FimH complex after mixing with DsF exactly according to the protocol described above for FimH·DsG and obtained in similar yields. Despite the non-natural interaction between FimH_P_ and DsF, the FimH^K12^·DsF complex was formed four times faster than the FimH^K12^·DsG complex at pH 7.0 and 37 °C, with a rate constant of 2.2±0.5 M^−1^ s^−1^. The FimH·DsF complex was stable against dissociation and unspecific aggregation.

### Determination of protein concentrations

Protein concentrations were measured via the specific absorbance at 280 nm, using the following extinction coefficients (FimH^K12^ and FimH^F18^ have identical extinction coefficients): FimC·FimH (59,090 M^−1^ cm^−1^), FimH_L_ (24,670 M^−1^ cm^−1^), FimH·DsG (35,090 M^−1^ cm^−1^) and FimH·DsF (36,580 M^−1^ cm^−1^). The concentrations of DsG and DsF were determined via their absorbance at 205 nm (42,650 and 49,700 M^−1^ cm^−1^, respectively).

### Synthesis of the fluorescent-labelled FimH ligand GN-FP-4

To a stirred solution of mannoside **1** (25 mg, 0.061 mmol)^55^ in dry dimethylformamide (DMF; 1 ml), N-hydroxysuccinimide (21 mg, 0.183 mmol) was added, followed by N,N′-dicyclohexylcarbodiimide (9.2 mg, 0.073 mmol). The mixture was stirred at room temperature for 2 h, then *N*-Boc-ethylendiamine (10.7 mg, 0.067 mmol) was added and the reaction was stirred for an additional 10 h. After cooling to 0 °C, the reaction mixture was diluted with water and concentrated. Chromatography on silica gel (CH_2_Cl_2_/MeOH) yielded 23 mg (0.042 mmol, 68%) of *tert*-butyl (3′-chloro-4′-(α-D-mannopyranosyloxy)-biphenyl-4-yl-carboxamido)ethyl)carbamate. This product was dissolved in CH_2_Cl_2_ (3 ml) and trifluoroacetic acid (TFA, 1 ml) was added. The solid dissolved during addition of TFA. After 10 min at room temperature the reaction was complete. The mixture was evaporated and excess TFA was removed in high vacuum. The intermediate *N*-(2-aminoethyl)-3′-chloro-4′-(α-D-mannopyranosyloxy)-biphenyl-4-carboxamide TFA salt (23 mg, 0.042 mmol, quant.) was used without purification in the next step. After dissolution in dry DMF (0.5 ml), triethylamine (12.8 mg, 0.127 mmol) was added. The mixture was cooled to 0 °C, then fluorescein isocyanate (14.8 mg, 0.038 mmol) was added and the mixture was stirred for 3 h in the dark. After the addition of water, DMF was removed azeotropically, the residue dissolved in MeOH/10% acetic acid and evaporated. Chromatography on silica gel (CH_2_Cl_2_/MeOH) yielded compound **2**, contaminated with triethylammonium acetate. Therefore, after dissolution in MeOH, 0.5 N HCl in MeOH was added, the mixture evaporated and chromatographed on silica gel to yield pure compound GN-FP-4 ((3′-Chloro-*N*-(2-(3-(3′,6′-dihydroxy-3-oxo-3*H*-spiro[isobenzofuran-1,9′-xanthen]-5-yl)-thioureido)ethyl)-4′-(α-D-mannopyranosyloxy)-biphenyl-4-carboxamide)^58^ (15 mg, 47%). [α]_D_^20^+12.1 (*c* 0.3, MeOH); ^1^H NMR (500 MHz, CD_3_OD): *δ*=8.12 (s, 1H), 7.92 (d, *J*=8.3 Hz, 2H, Ar–H), 7.70 (dd, *J*=5.0, 13.1 Hz, 2H, Ar–H), 7.64 (d, *J*=8.3 Hz, 2H, Ar–H), 7.54 (dd, *J*=2.2, 8.6 Hz, 1H, Ar–H), 7.46 (d, *J*=8.7 Hz, 1H, Ar–H), 7.09 (d, *J*=8.2 Hz, 1H, Ar–H), 6.74 (s, 2H), 6.69 (d, *J*=1.4 Hz, 2H, Ar–H), 6.55 (d, *J*=8.4 Hz, 2H, Ar–H), 5.63 (d, *J*=1.3 Hz, H-1), 4.15 (dd, *J*=1.8, 3.1 Hz, H-2), 4.03 (dd, *J*=3.4, 9.5 Hz, H-3), 3.94 (s, 2H, CH_2_), 3.86-3.64 (m, 6H, H-4, H-5, H-6, CH_2_); ^13^C NMR (126 MHz, CD_3_OD): *δ*=153.21, 143.84, 136.41, 129.66, 129.18, 127.76, 127.70, 125.37, 118.64, 103.62 (Ar-C), 100.75 (C-1), 76.00 (C-5), 72.41 (C-3), 71.86 (C-2), 68.24 (C-4), 62.69 (C-6), 40.76 (CH_2_); ESI–MS: *m/z*: Calculated for C_42_H_37_ClN_3_O_12_S [M+H]^+^: 842.2, found: 842.2.

### Fluorescence spectroscopy

Fluorescence emission spectra of FimH variants were recorded between 300 and 450 nm (excitation at 280 nm) at 25 °C in 1.0 × 0.4-cm quartz cuvettes on a QM 7/2003 spectrofluorimeter (PTI, USA) equipped with a magnetic stirrer. Protein concentrations were 1−2 μM in 20 mM MOPS–NaOH pH 7.4. Fluorescence spectra of GN-FP-4 (*ɛ*_495 nm_=54,900 M^−1^ cm^−1^) were recorded between 500 and 650 nm (excitation at 497 nm) in the same buffer.

### Kinetics of HM binding to FimH·DsG

The rate constants of binding (*k*_on_) and dissociation (*k*_off_) for the complex between FimH·DsG and HM were measured at 25 °C in 20 mM MOPS–NaOH pH 7.4 in a SX20 stopped-flow instrument (Applied Photophysics, UK). A constant FimH·DsG concentration of 1 or 2 μM was used. FimH·DsG was mixed with different concentrations of HM (2−100 μM), and binding was monitored by the increase in fluorescence above 320 nm (excitation at 280 nm). The fluorescence traces were globally fitted with *Dynafit*^59^ according to a second-order binding and first-order dissociation reaction. As an additional control, the fluorescence amplitudes of the individual reactions were plotted against the total HM concentration and fitted according to equation (2). The deduced dissociation constants reproduced the *K*_d_ values obtained with equilibrium titration within experimental error.

### Equilibrium titration of FimH·DsG with HM

The binding equilibrium between FimH·DsG and HM was followed at 25 °C in 20 mM MOPS–NaOH pH 7.4 on a QM 7/2003 spectrofluorometer (PTI) by the increase in fluorescence at 320 nm on HM binding (excitation at 280 nm). Measurements were performed with a stirred 1 × 0.4-cm quartz cuvette. The concentration of FimH·DsG was kept constant at 2 μM and the concentration of HM was varied between 0 and 200 μM. The samples were equilibrated overnight, and their fluorescence intensities were recorded for 30 s and averaged. The fluorescence intensities were plotted against total HM concentration and fitted according to equation (2)


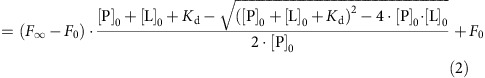


where *F* is the monitored fluorescence signal, *F*_0_ is the fluorescence signal in absence of ligand, *F*_*∞*_ is the fluorescence signal at full saturation with ligand, *K*_d_ is the dissociation constant, [P]_0_ is the total concentration of FimH·DsG and [L]_0_ is the total concentration of HM.

### Equilibrium titration of FimH_L_ with GN-FP-4

The binding equilibrium between FimH_L_ and GN-FP-4 at 25 °C in 20 mM MOPS–NaOH pH 7.4, supplemented with 0.001% Tween 20 to prevent unspecific adsorption effects at nanomolar concentrations, was recorded by the decrease in GN-FP-4 fluorescence at 520 nm (excitation at 497 nm). Measurements were performed with a stirred 1 × 0.4-cm quartz cuvette. The concentration of GN-FP-4 was kept constant at 1.0 or 2.0 nM and the concentration of FimH_L_ was varied between 0 and 10 nM. The samples were equilibrated overnight, and their fluorescence intensities at 520 nm were recorded for 30 s and averaged. The experimental data were fitted according to equation (2).

### Displacement of HM from the FimH_L_ by GN-FP-4

The rate constant of dissociation (*k*_off_) for HM from FimH_L_ at 25 °C in 20 mM MOPS–NaOH pH 7.4 was measured indirectly by binding of excess GN-FP-4 to FimH_L_ after dissociation of HM, recorded with the decrease in GN-FP-4 fluorescence at 520 nm (excitation at 497 nm). A mixture of FimH_L_ and HM (3 μM each), pre-incubated for at least 18 h, was mixed with different amounts of excess GN-FP-4 (final concentrations: 10−40 μM), and GN-FP-4 fluorescence was recorded every 10 min for 10 s, averaged and the data fitted according to first-order kinetics. The obtained rate constants were independent of GN-FP-4 concentration and thus identical to the dissociation rate of HM from FimH_L_.

### Determination of the FimH_L_·HM dissociation constant

The affinity of FimH_L_ for HM at 25 °C in 20 mM MOPS–NaOH pH 7.4 was determined by the competition between HM and GN-FP-4 for binding to FimH_L_. A mixture of FimH_L_ and GN-FP-4 (1 μM each) was incubated with different concentrations of HM (10–3.2 mM) and incubated for at least 18 h. The displacement of GN-FP-4 by HM was recorded on by the decrease in the fluorescence polarization at 528±20 nm (excitation at 485±20 nm) on a microplate reader (Biotek, USA), using flat black-bottom 96-well microtitre plates (Greiner, Austria). The fluorescence polarization data were fitted with *Dynafit*^59^ according to an equilibrium competition mechanism, with the total concentrations of FimH_L_, GN-FP-4 and HM (variable) and the respective *K*_d_ of GN-FP-4 (Table 2) as input, and *K*_d_ of HM and the fluorescence polarization at zero and infinite HM concentration as open parameters.

### Crystallization of FimH variants

All crystallization experiments were performed at 4 °C with the sitting drop vapour diffusion method. For crystallization, FimH^F18^·DsG and FimH^K12^·DsG (0.1−0.2 μl, 15 mg ml^−1^ in H_2_O) was mixed with 0.1−0.2 μl of precipitant (25% (w/v) polyethylene glycol (PEG) 3350, 0.2 M magnesium chloride, 0.1 M BisTris-HCl pH 5.5 at 4 °C. Crystals of FimH^F18^·DsG and FimH^K12^·DsG grew within 4−6 weeks and are of the space group C2, with one molecule per asymmetric unit. FimH^K12^·DsG crystals in space group P1 grew at 0.2 M Na Malonate, 20% PEG3350 within 2 months at 4 °C. For crystallization of the FimH^F18^·DsG·HM complex, a threefold excess over FimH·DsG was used (protein concentration and protein/precipitant ratios were as described for FimH·DsG). Crystals of the space group P2_1_3 appeared after 1 month in 30% (v/v) 2-Methyl-2,4-pentanediol, 0.1 M sodium cacodylate, 0.2 M magnesium acetate pH 6.5 at 4 °C. FimH^K12^·DsF·HM crystals appeared after 2 months in 30% w/v PEG 5000, 0.1 M 2-(*N*-morpholino)ethanesulfonic acid (MES) monohydrate, 0.2 M ammonium sulfate pH 6.5 at 20 °C (2.5-fold excess of ligand over protein). FimH_L_^F18^·HM crystallized in 17% PEG 2000 MME, 0.1 M HEPES–NaOH pH 7.5 at 4 °C. FimH_L_^K12^·HM crystallized in 1.5 M (NH4)_2_SO_4_, 0.2 M Na acetate pH 5.5 at 20 °C.

### Crystallographic data collection

All crystals, except for FimH^F18^·DsG·HM, were cryo-preserved by the addition of ethane-1,2-diol to a final concentration of 20% (v/v). The precipitant solution used for the crystallization of FimH^F18^·DsG·HM already contained 30% (v/v) methyl-2,4-pentanediol, which acts as cryoprotectant. Crystals were flash-cooled in liquid nitrogen. All measurements were carried out at the SLS beamline X06DA and X06SA (Swiss Light Source, Paul Scherrer Institute, Switzerland) at 100 K. All data were integrated, indexed and scaled using the XDS software package^60^ (5% of the reflections were set aside as test set). Data collection statistics are summarized in Table 1.

### Crystallographic structure determination

All structures were solved by molecular replacement using structures of isolated FimH_L_ (AA1-158, PDB ID: 3MCY^18^, and the pilin domain of FimC·FimH (AA160-297, PDB ID: 1QUN^24^, as search models with the programme Phaser^61^). Model building and structure refinement were performed with Coot (ref. 62) and PHENIX (ref. 63). Twelve out of thirteen residues could be built for the FimG donor strands in the crystal structures, and only the C-terminal lysine residue had weak electron density. Refinement statistics are summarized in Table 1.

### Molecular dynamics simulations

Four molecular systems were prepared for FimH^F18^. The first system was constructed using the **A**_**bound**_ state of FimH^F18^·DsG·HM (Supplementary Fig. 3a,b) and the second system is equivalent but HM was removed (Supplementary Fig. 3c,d). The third system contains only the FimH_L_ and HM from the **A**_**bound**_ X-ray structure (Supplementary Fig. 3e,f). The fourth system was prepared for FimH_L_ and HM based on the **S**_**bound**_ state in the FimH^K12^·DsF·HM crystal structure (Supplementary Fig. 3g,h).

The CHARMM-GUI web server^64^ was used to prepare the molecular systems, which were solvated with TIP3 water molecules and ionized with 50 mM NaCl. Each system contains between 50,000 and 60,000 atoms. All simulations were performed with the NAMD simulation package (version 2.9) (ref. 65). The CHARMM36 force field was used for the protein, and parameters for HM were generated using the CHARMM General Force Field programme (version 0.9.7 beta). Electrostatic interactions were calculated using the particle-mesh Ewald method^66^ with a grid spacing of 1 Å. The cutoff for the van der Waals interactions was taken at 12 Å with a switching function used after 10 Å. Time step for the integration of dynamics was 2 fs. Simulations were performed in an isothermal–isobaric ensemble, with a pressure of 1 atm and a temperature of 300 K.

### Cell tracking on mannose–BSA-coated surfaces

The *E. coli* KB18 strain^67^ was kindly provided by Professor Evgeni Sokurenko and served as host for the generation of recombinant strains. KB18 contains the pPKL114 plasmid^57^, which encodes the whole *fim* operon with a translational stop linker upstream of the *fimH* gene. KB18 was co-transformed with the pGB2-24 plasmid, which was isolated from the ELT115 strain and encodes *fimH*^J96^ (kindly provided by Professor Evgeni Sokurenko). Single-nucleotide point mutations were introduced in *fimH*^J96^ using overlap extension PCR following standard molecular techniques to obtain *fimH*^F18^ and *fimH*^F18^-Ala188Asp. The PCR products were cloned into pGB2-24 by the ApaLI and SphI sites, and KB18 was transformed with the resulting plasmid.

*E. coli* strains were grown from frozen stocks in LB medium supplemented with antibiotics (100 μg ml^−1^ ampicillin and 25 μg ml^−1^ chloramphenicol) until late log phase (OD_600_ of 1.0–1.2) and diluted to an OD_600_ of 0.01 before movie acquisition.

Cell culture dishes (35 mm, Corning Inc., Corning, NY) were incubated with 50 μl of 50 μg ml^**−**1^ 1M-BSA in 0.02 M bicarbonate buffer for 75 min at 37 °C. The dishes were then washed three times and quenched with 0.1% PBS–BSA to remove unbound 1M-BSA and block remaining sites on the plastic surface to prevent nonspecific binding of bacteria. Controls were prepared by treating cell culture dishes with 0.1% PBS–BSA only. The bacterial suspension was added to the cell culture dishes for microscopy studies.

Cell tracking was carried out at room temperature under static conditions. A bacterial suspension of 50 μl in the late logarithmic growth phase was placed onto the cell culture dishes (diluted to OD_600_ of 0.01), and a cover slide was placed on top. The delay between sample placement and start of the movie acquisition was about 1 min. Time-lapse movies were recorded with a × 20 phase contrast objective using a CMOS digital camera (The Imaging Source Europe, Bremen, Germany) mounted on a Nikon Ti Eclipse inverted microscope and using the NIS Elements Basic Research software (Nikon, Zurich, Switzerland). Phase contrast images in an ∼5-μm-thick surface layer were taken at four to five frames per second over 5 min. The dead time of movie acquisition was ∼1 min. The resulting images were segmented by creating a projection of the average intensities over all frames to remove the background and by subsequent thresholding using the Maximum Entropy method in Fiji^68^ to obtain binary images (examples shown in Supplementary Movies 1 and 2). The segmented movies were imported into Imaris (Bitplane, Zurich, Switzerland) and tracked through the autoregressive algorithm. A time filter was applied to exclude all tracks with a length below 15 s. Tracks longer than 15 s were reviewed individually and edited manually, if necessary. Five to seven independent movies were recorded for each experimental set-up: FimH^F18^ or FimH^F18^-Ala188Asp on 1M-BSA-coated dishes and FimH^F18^ and FimH^F18^-Ala188Asp on BSA-coated cell culture dishes. *E. coli* piliated with FimH^F18^ or FimH^F18^-Ala188Asp binding to 1M-BSA in the absence (1,815 and 1,283 individual tracks, respectively) and in the presence of 200 μM HM (1,175 and 1,071 individual tracks, respectively) were analysed respectively. For *E. coli* piliated with FimH^F18^ or FimH^F18^-Ala188Asp binding to BSA 1,314 and 1,065 individual tracks, respectively, were analysed. Bacteria with a speed of <0.5 μm s^−1^ were classified as attached, all other bacteria were classified as mobile. Owing to limitation in the spatial and temporal resolution of movie acquisition, we did not further subdivide bacterial swimming into motility behaviours as ‘rolling'^29^,^53^, ‘roaming', ‘orbiting' and so on. The individual cell tracks were classified into four classes: no motility change during observation (pre-attached or mobile), transient attachment, permanent attachment and permanent detachment. For FimH^F18^ on 1M-BSA surfaces, 13.9% (251 out of 1,815 tracks) of all tracks showed a single transient attachment event (Supplementary Fig. 7d). In total, 67 out of the 251 bacteria that underwent a first transient adhesion attached and detached from the surface a second time. For these cells, the average time between detachment and re-attachment was only 13.5 s (Supplementary Fig. 7e), suggesting that re-binding may be favoured by proximity to the surface as compared with the initial attachment. The mean velocity on 1M-BSA, as compared with BSA-coated surfaces, was reduced for both FimH^F18^-piliated (4.2 and 7.4 μm s^−1^, respectively) and FimH^F18^–Ala188Asp-piliated bacteria (3.5 and 8.1 μm s^−1^, respectively; Supplementary Fig. 7a,b). This reduction of the mean velocity originates from two different phenomena: in FimH^F18^-piliated cells it is caused by a change from fast swimming to a slower mode of motion (Supplementary Fig. 7b; Supplementary Movie 1), which is consistent with bacterial surface rolling due to weak, short-lived mannose-based interactions^29^,^53^. In contrast, for FimH^F18^–Ala188Asp-piliated bacteria, the reduction of the mean velocity results from an increase in the fraction of adherent cells on 1M-BSA compared with BSA (see main text). In the presence of 200 μM HM, the mean velocity on 1M-BSA is increased for both FimH^F18^-piliated (6.5 μm s^−1^) and FimH^F18^–Ala188Asp-piliated bacteria (5.9 μm s^−1^; Supplementary Fig. 7a) and transient and permanent attachment is reduced by 75% and 85%, respectively (Fig. 6 and Supplementary Fig. 7c).

## Additional information

**Accession codes:** The atomic coordinates and structure factors have been deposited in the Protein Data Bank under the accession codes 4XO8, 4XO9, 4XOA, 4XOB, 4XOC, 4XOD, 4XOE.

**How to cite this article:** Sauer, M. M. *et al.* Catch-bond mechanism of the bacterial adhesin FimH. *Nat. Commun.* 7:10738 doi: 10.1038/ncomms10738 (2016).

## Supplementary Material

Supplementary Information Supplementary Figures 1-7, Supplementary Note 1 and Supplementary References.

Supplementary Movie 1 Time-lapse analysis of FimHF18 (Abound) piliated E. coli cells on a mannosylated surface. Images were recorded over five minutes, segmented, background subtracted and converted into binary files by subsequent thresholding. The supplementary video is accelerated six-fold. In the first part (1–16 s) raw images are shown, the second part (16–26 s) is splitted into raw images (left) and binary images (right), the third part (26–42 s) shows the processed images.

Supplementary Movie 2 Time-lapse analysis of FimHF18-Ala188Asp (Sbound state favored relative to FimHF18) piliated E. coli cells on a mannosylated surface. Images were recorded over five minutes, segmented, background subtracted and converted into binary files by subsequent thresholding. The supplementary video is accelerated six-fold. In the first part (1–16 s) raw images are shown, the second part (16–26 s) is splitted into raw images (left) and binary images (right), the third part (26–42 s) shows the processed images.

## Figures and Tables

**Table 1:** Data collection and refinement statistics

	**FimH**^**F18**^**·DsG**	**FimH**^**K12**^**·DsG**	**FimH**^**K12**^**·DsG·HM**	**FimH**^**F18**^**·DsG·HM**	**FimH**^**K12**^**·DsF·HM**	**FimH**_**L**_^**F18**^**·HM**	**FimH**_**L**_^**K12**^**·HM**
*Data collection*							
Space group	C 1 2 1	C 1 2 1	P 1	P 21 3	I 21 21 21	C 2 2 21	P 21 21 21
Cell dimensions							
*a, b, c* (Å)	99.3, 35.5, 72.8	99.5, 35.6, 72.9	56.5, 77.6, 78.1	128.4, 128.4, 128.4	94.5, 147.1, 250.8	140.1, 176.1, 28.3	63.0 68.4 95.9
α, β, γ (°)	90, 105, 90	90, 105, 90	101.5, 111.1, 96.3	90, 90, 90	90, 90, 90	90, 90, 90	90, 90, 90
Resolution (Å)	70–1.14 (1.21–1.14)	48–1.14 (1.2–1.14)	52–2.54 (2.63–2.54)	128–2.4 (2.5–2.4)	48.1–3.0 (3.19–3.0)	54–1.42 (1.47–1.42)	52–1.7 (1.76–1.7)
*R*_merge_	0.039 (0.760)	0.038 (0.691)	0.161 (0.859)	0.207 (2.424)	0.427 (1.741)	0.121 (1.242)	0.129 (1.278)
CC_1/2_	100.0 (68.0)	100.0 (84.2)	99.2 (82.2)	99.8 (56.3)	95.8 (46.7)	100.0 (71.0)	99.9 (57.7)
*I*/σ*I*	16.6 (1.6)	17.8 (2.8)	8.79 (2.0)	18.3 (1.6)	14.4 (1.2)	17.9 (2.0)	15.1 (1.7)
Completeness (%)	97.6 (83.5)	94.7.0 (81.5)	90.7 (89.4)	99.9 (99.9)	99.9 (98.7)	98.9 (94.4)	96.9 (79.3)
Redundancy	3.1 (2.5)	3.2 (2.5)	3.4 (3.5)	20.4 (20.0)	6.6 (4.1)	16.9 (13.4)	11.5 (6.3)
							
*Refinement*							
Resolution (Å)	70.1–1.14	47.9–1.14	52.6–2.54	74.1–2.4	48.13–3.0	54–1.42	52.6–1.7
No. reflections	273774 (87283)	277806 (85217)	122641 (11994)	567915 (28396)	172553 (35275)	83287 (6222)	518524 (23173)
*R*_work_/*R*_free_	0.165/0.185	0.153/0.175	0.227/0.276	0.155/0.179	0.220/0.251	0.149/0.175	0.171/0.196
No. atoms	3102	3086	8855	2866	8751	3099	3232
Protein	2590	2590	8690	2558	8652	2427	2407
Ligand/ion	—	—	—	19	86	57	38
Water	512	496	165	284	13	596	471
B-factors							
Protein	13.8	13.3	43.7	33.5	53.2	13.2	21.3
Ligand/ion	—	—	39.7	21.3	32.3	23.0	20.7
Water	26.2	23.6	22.4	39.1	25.9	28.2	40.6
R.m.s deviations							
Bond length (Å)	0.009	0.01	0.019	0.013	0.004	0.015	0.004
Bond angles (°)	1.29	1.45	1.78	1.38	0.82	1.62	1.02

*Values in parenthesis are for the highest resolution shell.

**Table 2:** Kinetics and thermodynamics of HM binding to FimH_L_ or FimH·DsG at pH 7.4 and 25 °C.

**Protein**	***k***_**on**_ **(M**^**−1 **^**s**^**−1**^**)**	***k***_**off**_ **(s**^**−1**^**)**	***k***_**off**_**/*****k***_**on**_ **(M)**^(a)^	***K*_d_ (amplitude analysis; M)**^(b)^	***K*_d_ (equilibrium titration; M)**^(c)^
FimH_L_^K12^	1.8±0.6 × 10^5(d)^	2.0±0.4 × 10^**−**4^	n.a.	n.a.	1.1±0.1 × 10^**−**9(e)^
FimH^K12^·DsG	5.0±0.1 × 10^6^	2.2±0.1 × 10^1^	4.3±0.1 × 10^**−**6^	4.2±0.3 × 10^**−**6^	3.6±0.3 × 10^**−**6^
FimH_L_^F18^	1.2±0.4 × 10^5(d)^	3.5±0.8 × 10^**−**4^	n.a.	n.a.	3.0±0.2 × 10^**−**9(e)^
FimH^F18^·DsG	4.9±0.1 × 10^6^	5.8±0.1 × 10^1^	1.2±0.04 × 10^**−**5^	1.2±0.1 × 10^**−**5^	9.9±1.5 × 10^**−**6^

HM, *n*-heptyl α-D-mannoside.

The rate constants *k*_on_ and *k*_off_ were determined from experiments as shown in Figs 4 and 5. The *K*_D_ values for the complex formation between HM constructs and the different FimH constructs were determined (a) from the ratio of rate constants (*k*_off_/*k*_on_), (b) from the analysis of the amplitudes as in Fig. 4d and (c) from equilibrium titration as in Fig. 4b. (d) *k*_on_ values were calculated with *k*_off_ and *K*_d_. (e) Values of *K*_d_ were obtained from competition equilibria with the fluorescent mannoside GN-FP-4 (Fig. 5a) and the following *K*_d_ values of GN-FP-4 binding determined in Supplementary Fig. 5: FimH_L_^K12^: *K*_d_=7.0±0.1 × 10^−11 ^M; FimH_L_^F18^: *K*_d_=1.8±0.2 × 10^−10 ^M.

**Table 3:** Comparison of HM binding by variants of FimH_L_ versus FimH·DsG.

**FimH variant**	***k***_**on**_ **(FimH**·**DsG)/*****k***_**on**_ **(FimH**_**L**_**)**	***k***_**off**_ **(FimH**·**DsG)/*****k***_**off**_ **(FimH**_**L**_**)**	***K*_d_ (FimH**·**DsG)/*K*_d_ (FimH**_**L**_**)**
K12	28	110,000	3,300
F18	41	170,000	3,300

HM, *n*-heptyl α-D-mannoside.
