# Supplementary material for: Catch-bond mechanism of the bacterial adhesin FimH
Source: Nat Commun. 2016 Mar 7;7:10738. doi: 10.1038/ncomms10738 (PMC4786642; doi:10.1038/ncomms10738)
Supplement: Supplementary Information — Supplementary Figures 1-7, Supplementary Note 1 and Supplementary References. [file ncomms10738-s1.pdf]

Supplementary Figures

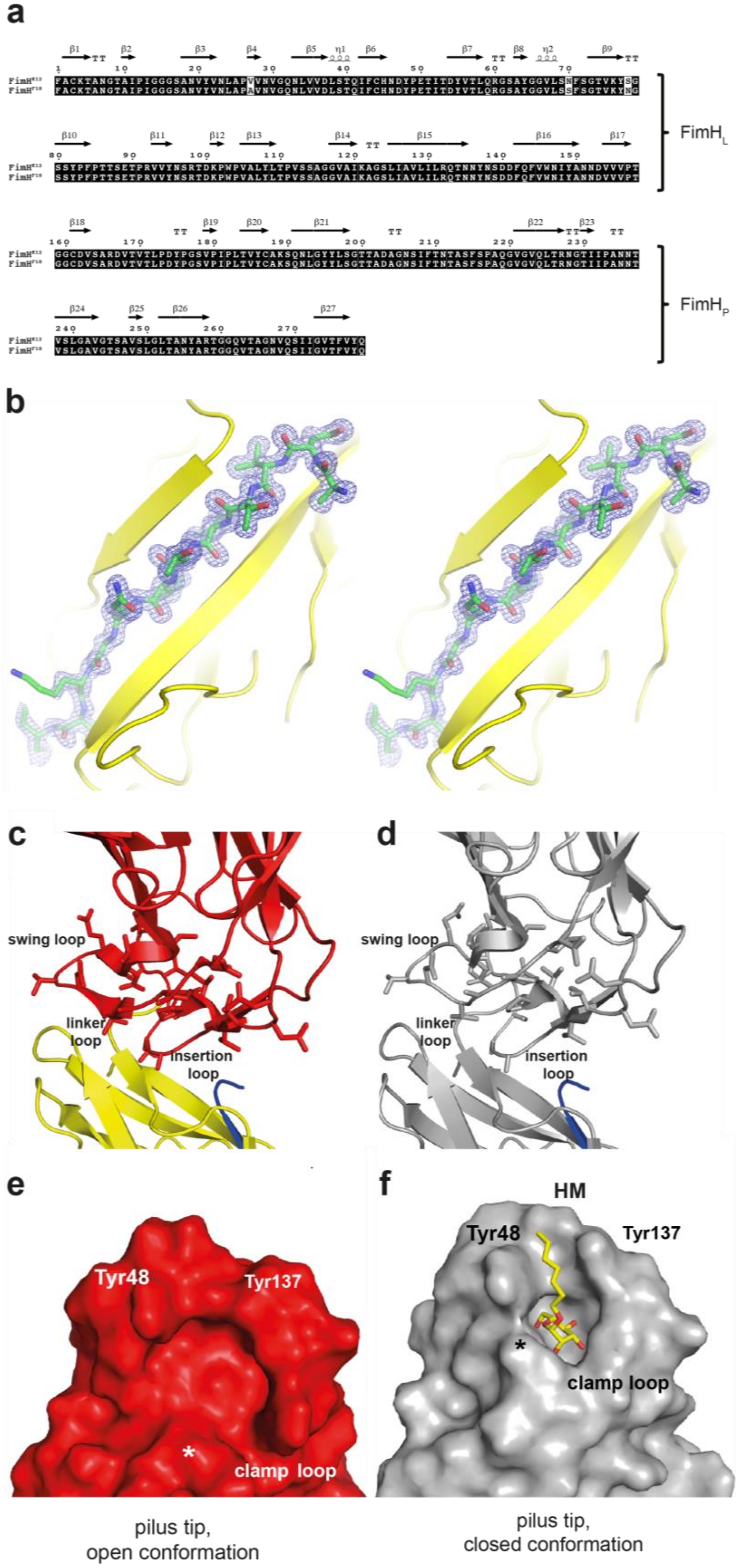

Supplementary Figure 1 Analysis of FimH sequence and conformational states. (**a**) Sequence variations between $FimH^{K12}$ and $FimH^{F18}$. Sequence alignment of FimH from the nonpathogenic *E. coli* strain K12 and the *E. coli* strain F18. (**b**) Stereo view of the electron density around the DsG peptide (stick representation) bound to $FimH^{F18}$·DsG. The 2FoFc map is shown in a radius of 2 Å around atoms belonging to the DsG peptide at 1.5σ contour level. (**c,d**) Comparison of the inter-domain region of FimH in the binary complex and $FimH^{F18}$·DsG (**A_{free}**) and of unliganded FimH in the context of the tip fibrillum. Enlarged view on the domain interface of $FimH^{F18}$·DsG (**b**) and the tip structure (**c**) (3JNW.pdb). (**e**,f) Closing of the mannose binding pocket upon substrate binding. Surface representation of the FimH lectin domain in (**e**) the open conformation of $FimH^{F18}$·DsG (**A_{free}**, red) and (**f**) the closed conformation in $FimH^{F18}$·DsG·HM (**A_{bound}**, grey). The tip of the clamp loop is indicated in both structures with an asterisk. The ligand HM is shown in stick representation (carbon atom: yellow; oxygen atom: red)

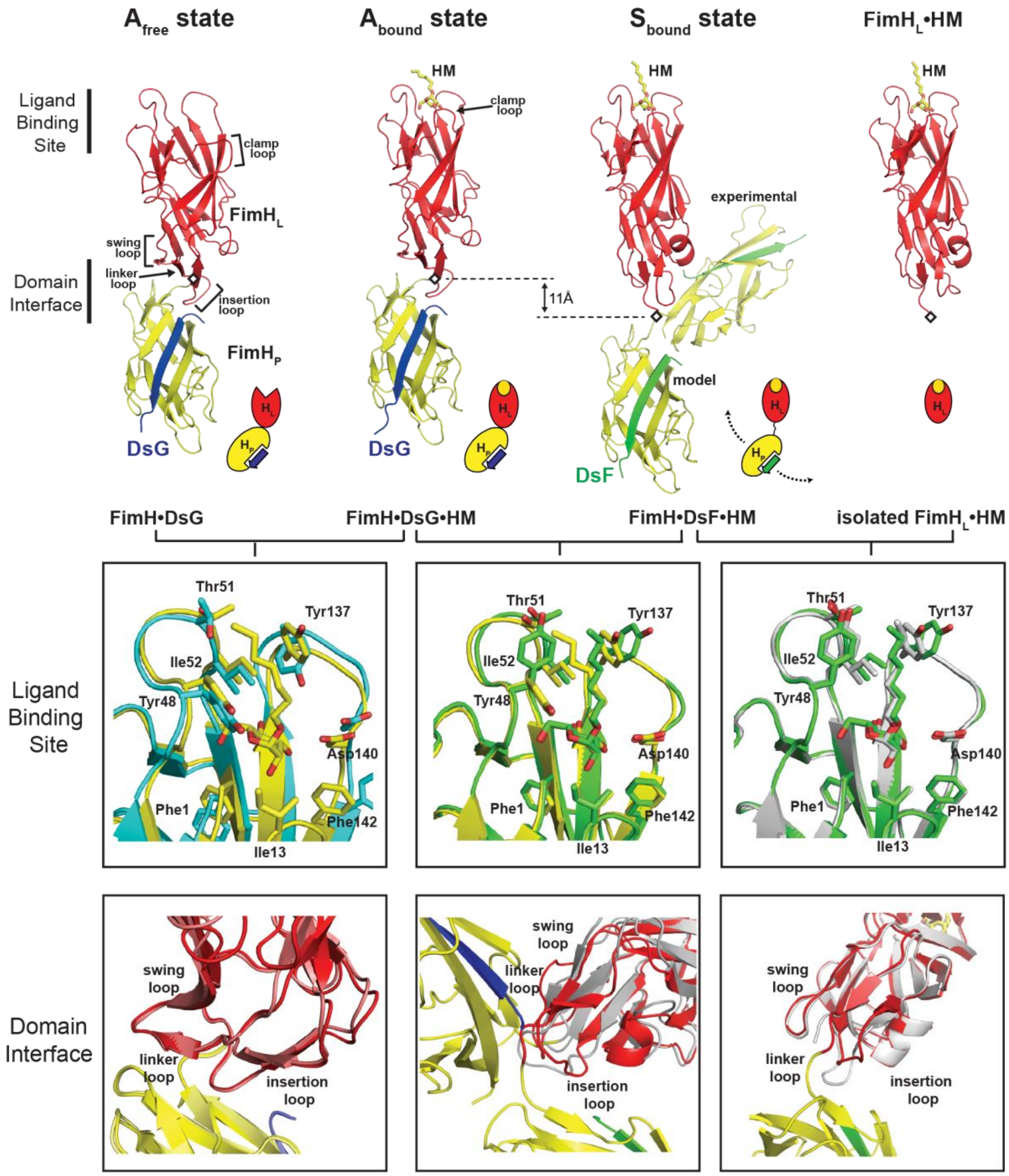

Supplementary Figure 2 Crystallographic analysis of the FimH ligand-binding site and the inter-domain interface of FimH in different conformational states. Top panel: Ribbon representation of the $\mathbf{A_{free}}$, $\mathbf{A_{bound}}$, $\mathbf{S_{bound}}$ state and $FimH_L$·HM represented by individual crystal structures. A schematic representation for each conformational state of FimH, similar to Fig. 1a and 2, is given. $FimH_L$, $FimH_P$, DsF and DsG are colored in red, yellow, green and blue, respectively. The experimentally *in crystallo* trapped orientation of $FimH_P$ in $FimH^{K12}$·DsF·HM and a modeled position (green) based on a hinge motion stretching around Gly157 is indicated. Lower panels: Pairwise comparison of the ligand-binding site and the inter-domain interface between conformational states as indicated. Upon ligand binding, the N-terminal $FimH_L$ residues preceding the clamp loop (residues

1–7) and the neighboring, large β-strand (aa. 142–152) slightly change their position. In addition, the loop segments 46–53 and 137–142 gently rearrange, resulting in a parallel alignment of the aromatic rings of Tyr48 and Tyr137, which sandwich the *n*-heptyl moiety of HM.

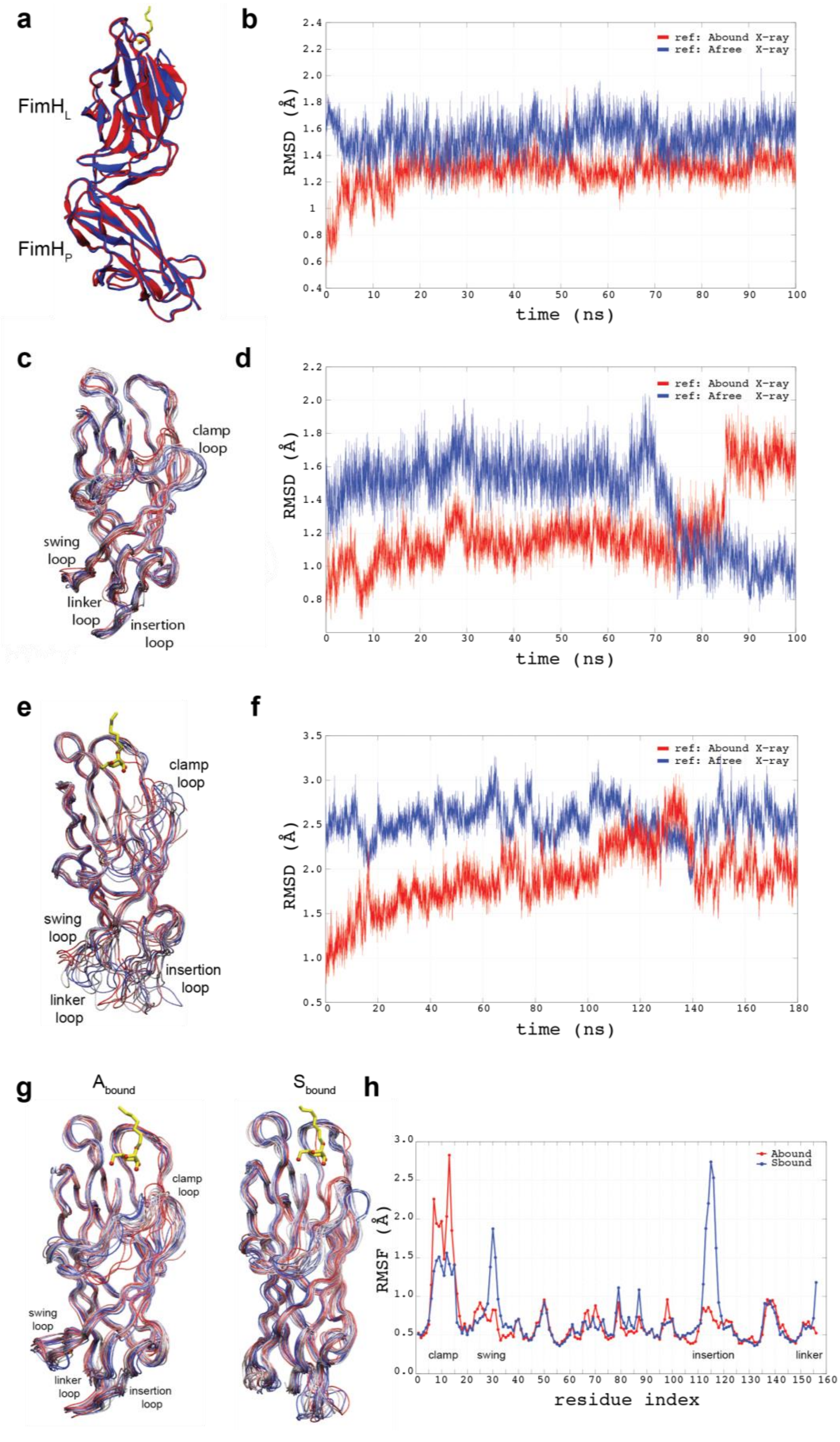
a
FimH$_L$
FimH$_P$
b
ref: Abound X-ray
ref: Afree X-ray
RMSD (Å)
time (ns)
c
clamp loop
swing loop
linker loop
insertion loop
d
ref: Abound X-ray
ref: Afree X-ray
RMSD (Å)
time (ns)
e
clamp loop
swing loop
insertion loop
linker loop
f
ref: Abound X-ray
ref: Afree X-ray
RMSD (Å)
time (ns)
g
A$_{bound}$
S$_{bound}$
clamp loop
swing loop
linker loop
insertion loop
h
Abound
Sbound
RMSF (Å)
clamp
swing
insertion
linker
residue index

Supplementary Figure 3 Molecular dynamics simulation of FimH conformational states. (**a, b**) Molecular dynamics simulation for the analysis of conformational stability of the $\mathbf{A_{bound}}$ state of FimH. (**a**) Structural comparison of snapshots at 0 ns (red) and 100 ns (blue) of a simulation run of the $\mathbf{A_{bound}}$ state with ligand. (**b**) rmsd against the X-ray template structures of the $\mathbf{A_{bound}}$ and $\mathbf{A_{free}}$ state during the MD simulation run of the $\mathbf{A_{bound}}$ state. (**c**) Structural comparison of snapshots of the MD simulation run for $\mathbf{A_{bound}}$ after removal of the ligand *in silico*. Snapshots are taken between 70 ns and 100 ns simulation time every 2 ns (colored from red to blue along simulation time). (**d**) rmsd against the X-ray template structures of the $\mathbf{A_{bound}}$ and $\mathbf{A_{free}}$ state during the MD simulation run of the $\mathbf{A_{bound}}$ state after removal of the ligand *in silico*. (**e, f**) Molecular dynamics simulation of the $\mathbf{A_{bound}}$ state of FimH upon *in silico* removal of $FimH_P$. (**e**) Structural comparison of snapshots every 20 ns along the 180 ns trajectory colored from red to blue along simulation time. (**f**) rmsd against $FimH_L$ in the X-ray template structures of the $\mathbf{A_{bound}}$ and $\mathbf{A_{free}}$ state during the MD simulation run of the $\mathbf{A_{bound}}$ state upon removal of $FimH_P$. (**g, h**) Dynamics of $FimH_L$ in molecular dynamics simulations of the $\mathbf{A_{bound}}$ and $\mathbf{S_{bound}}$ state of FimH (**g**) Structural comparison of snapshots every 5 ns along 100 ns colored from red to blue along simulation time. (**h**) Root mean square fluctuations of atomic coordinates in $FimH_L$ during the MD simulation runs of the $\mathbf{A_{bound}}$ and $\mathbf{S_{bound}}$ states of FimH. Structural fluctuations are increased by at least a factor of two in the swing, insertion and linker loop region, but also in the clamp loop region around residue 10.

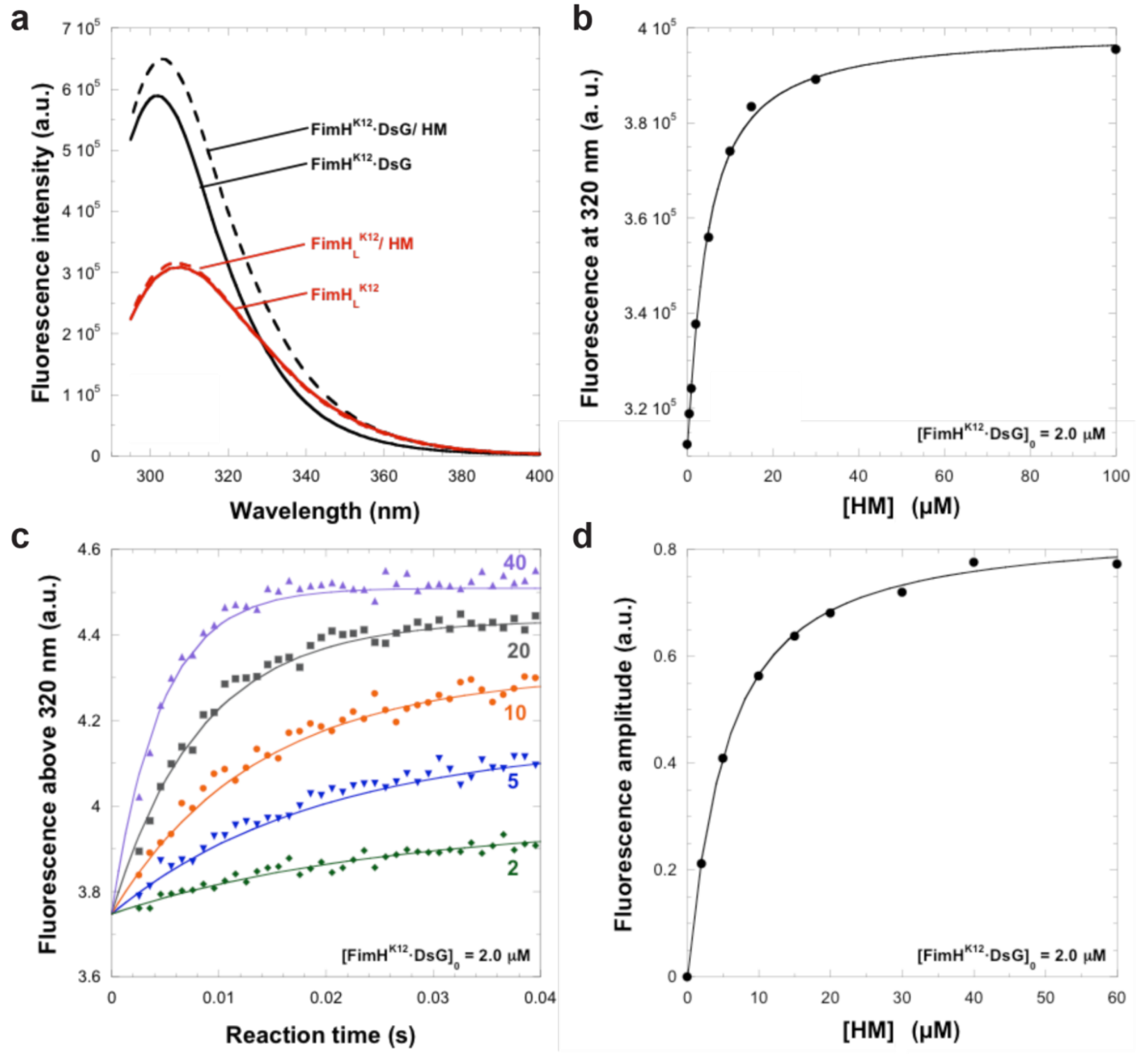

Supplementary Figure 4 HM binding and release by FimH^{K12}·DsG. (**a**) Fluorescence spectra (excitation at 280 nm) of FimH$_L^{K12}$ (2 μM) (red lines) and FimH^{K12}·DsG (2 μM) (black lines) in the absence (solid lines) or presence of 200 μM *n*-heptyl α-D-mannoside (HM) (dashed lines). (**b**) Equilibrium titration of FimH^{K12}·DsG (2 μM) with HM, recorded via the fluorescence increase at 320 nm. The total concentration of HM is plotted against the recorded fluorescence signal. Data were fitted (solid line) according to equation (2) (cf. experimental section) and yielded a K_d value of 3.6 ± 0.3 μM. (**c**) Stopped-flow fluorescence kinetics of HM binding to FimH^{K12}·DsG (2.0 μM), recorded via the fluorescence change above 320 nm. The HM concentration was varied between zero and 60 μM. Five representative traces are shown (HM concentrations are given in μM). The fluorescence traces were globally fitted according to a second-order binding and first-order dissociation reaction (solid lines) (Table 1). (**d**) Amplitudes of the reactions monitored in (**c**), plotted against the total HM concentration. Data were fitted (solid line) according to equation (2), yielding a K_d value of 4.2 ± 0.3 μM.

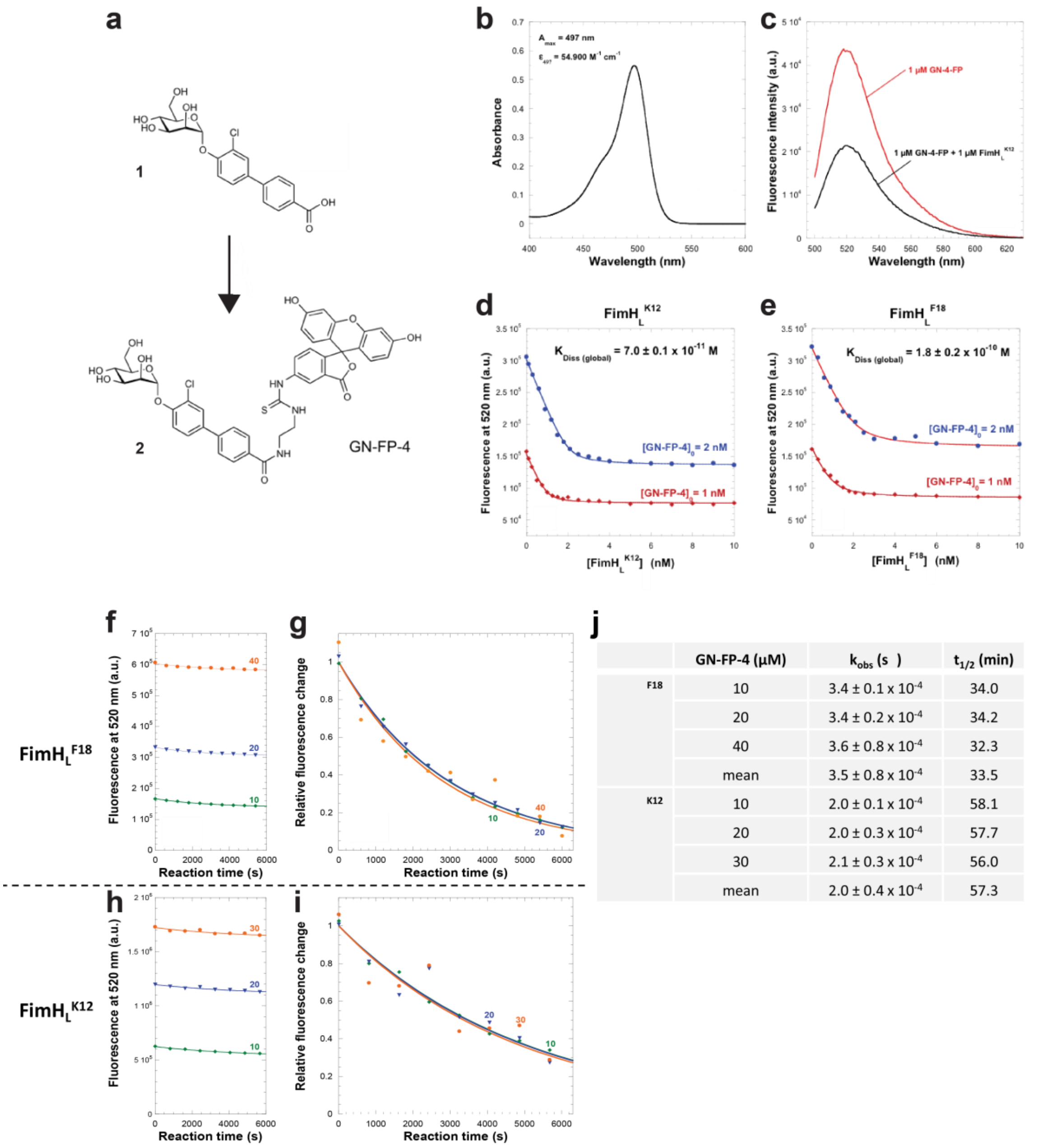

	GN-FP-4 (µM)	k_{obs} (s)	$t_{1/2}$ (min)
F18	10	3.4 ± 0.1 x 10^{-4}	34.0
	20	3.4 ± 0.2 x 10^{-4}	34.2
	40	3.6 ± 0.8 x 10^{-4}	32.3
	mean	3.5 ± 0.8 x 10^{-4}	33.5
K12	10	2.0 ± 0.1 x 10^{-4}	58.1
	20	2.0 ± 0.3 x 10^{-4}	57.7
	30	2.1 ± 0.3 x 10^{-4}	56.0
	mean	2.0 ± 0.4 x 10^{-4}	57.3

Supplementary Figure 5 Synthesis and spectroscopic properties of the fluorescent ligand GN-FP-4 and its application to determine ligand affinity and ligand binding kinetics. (**a**) Reaction conditions of GN-FP-4 synthesis: i) DIC, NHS, *N*-Boc-ethylenediamine, DMF, rt, 12 h; ii) TFA, DCM, rt, 10 min (68% over two steps), iii) fluorescein isothiocyanate (FITC), NEt_3, DMF, rt, 3 h (48%). (**b**) Absorbance spectrum of 10 µM GN-FP-4. (**c**) Fluorescence spectra of GN-FP-4 (1 µM) before (red line) and after (black line) addition of one molar equivalent of $FimH_L^{K12}$. The results, together with the high affinity of the $FimH_L^{K12}$·GN-FP-4 complex (0.08 nM), show that the GN-FP-4 fluorescence decreases about 2-fold upon binding. (**d**, **e**) Equilibrium titration of GN-FP-4 (1.0 nM) with $FimH_L^{K12}$ (**d**) or $FimH_L^{F18}$ (E) at pH 7.4 and 25 °C, recorded via the decrease in GN-FP-4 fluorescence at 520 nm upon binding to the FimH lectin domain (excitation at 497 nm). Titrations were performed at constant GN-FP-4 concentrations of

1 nM (blue circles) or 2 nM (red rhombs) and globally fitted according to equation 2 (solid lines). Samples contained 0.001% Tween 20 to prevent unspecific adsorption of GN-FP-4 to tubes or cuvettes. Determination of the rate constant of dissociation of HM from $FimH_L^{F18}$ (**f**,**g**) or $FimH_L^{K12}$ (**h**,**i**) by displacement of HM with GN-FP-4 (pH 7.4, 25 °C). In all experiments, the 1:1 complex between $FimH_L$ and HM (3 µM each) was mixed with excess GN-FP-4 (concentrations given in µM in F–I). As the kinetics of GN-FP-4 binding were independent of GN-FP-4 concentration (**g**,**h**,**i**), the rate-limiting step of GN-FP-4 binding was the dissociation of HM from $FimH_L$. The decrease in GN-FP-4 fluorescence thus coincided with the dissociation of the $FimH_L$·HM complex. (**f**,**h**) raw fluorescence data; (**g**,**i**), superimposed, normalized fluorescence kinetics. (j) Rate constants and half-lifes of HM association at different concentrations of excess GN-FP-4.

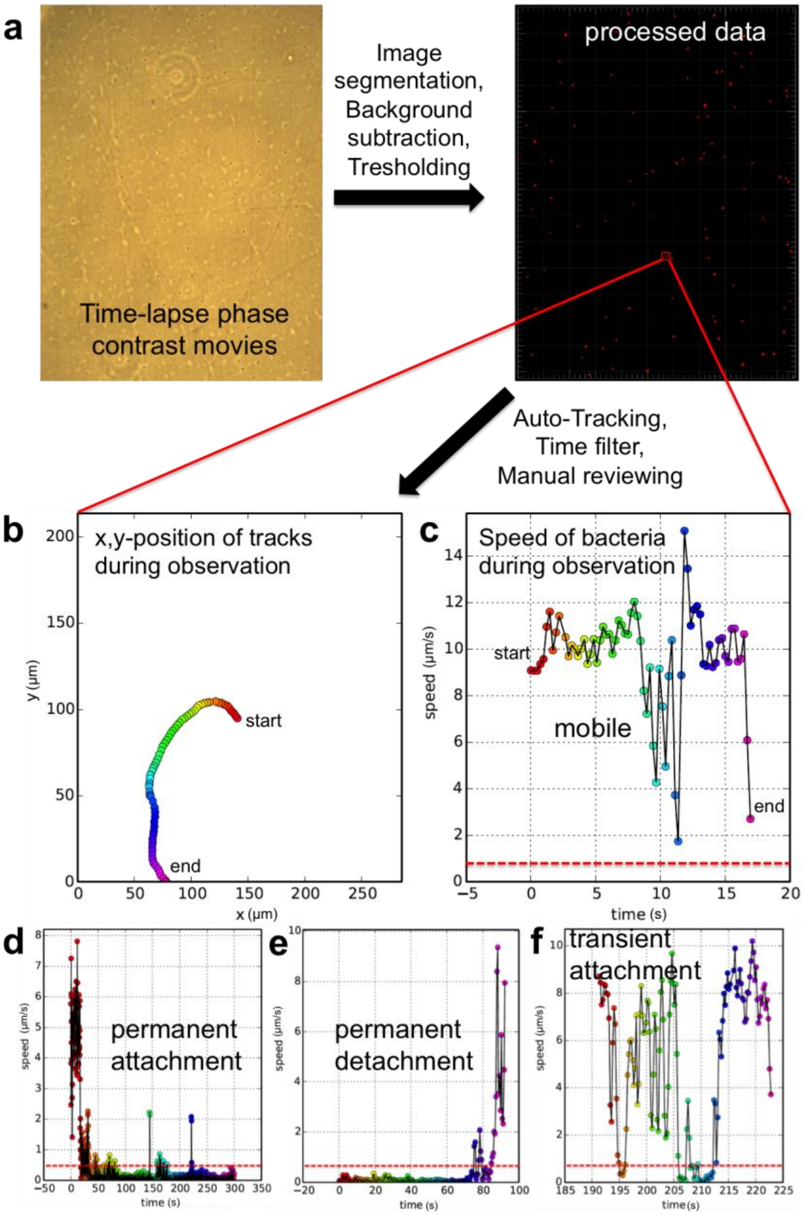

Supplementary Figure 6 Cell tracking data processing and analysis. (**a**) Time-lapse movies were recorded, with four to five frames per second over five minutes. The resulting images were segmented by creating a projection of the average intensities over all frames to remove the background and by subsequent thresholding binary images were obtained. Bacteria were tracked through an autoregressive algorithm and a time filter was applied, and only tracks >15 s were analyzed. (**b**) From the movies the x,y-position of the tracked bacteria during the time of observation was determined (left), rainbow color coded, with red indicating the starting and magenta the endpoint of observation. (**c**) The speed of the bacteria was extracted and plotted against the observation time. Bacteria with a speed of <0.5 μm s^{-1} were classified as attached (indicated by the red dotted line), all other as swimming. The tracks were classified in four classes: mobile (motility > 0.5 μm s^{-1}) (**c**), permanent attachment (**d**), permanent detachment (**e**), and transient attachment (**f**). The time point zero indicates the start of movie acquisition.

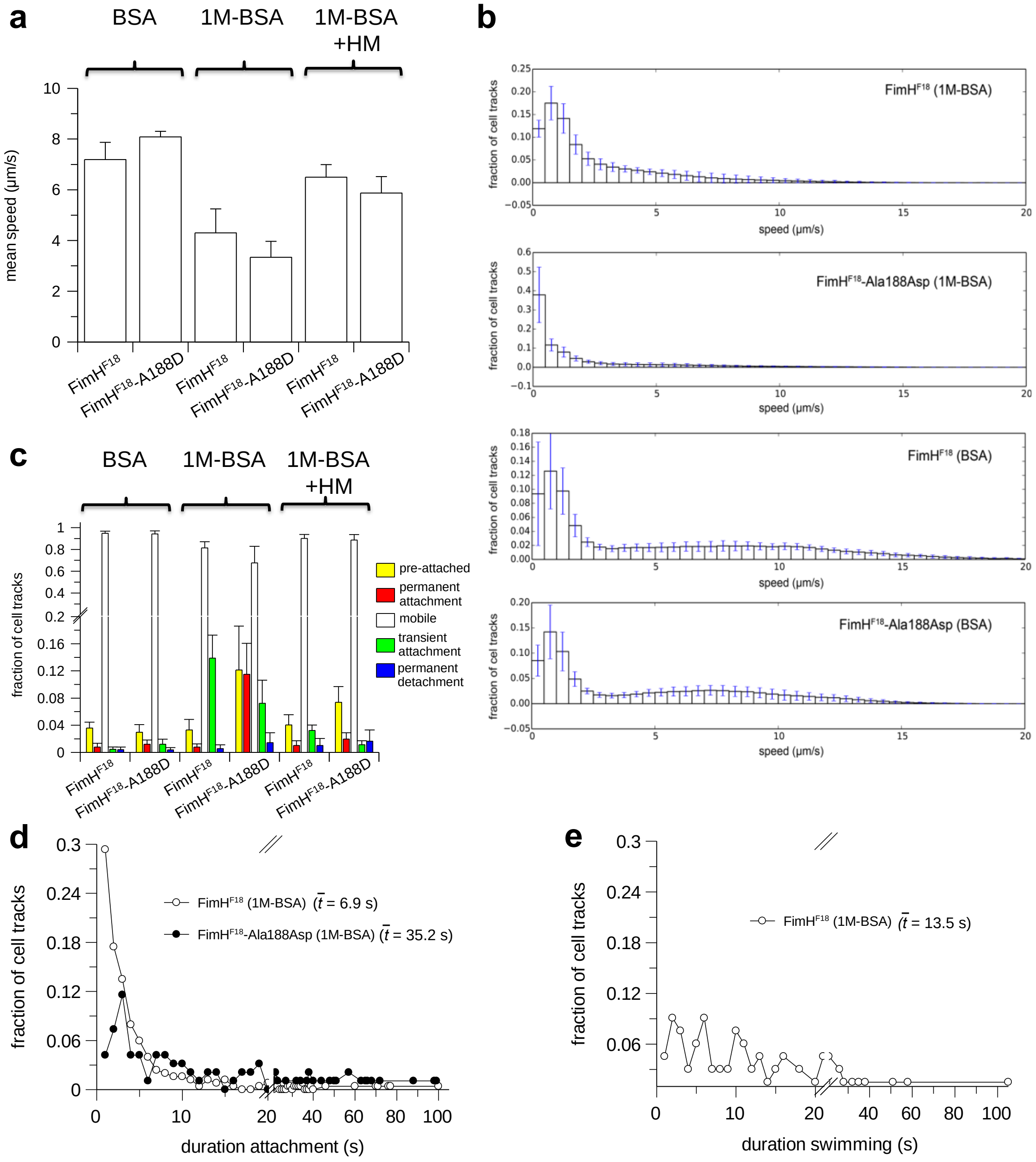

Supplementary Figure 7 Cell-tracking analysis of bacterial motility on and adhesion to mannosylated surfaces. Differences of FimH^{F18} and FimH^{F18}-Ala188Asp mediated adhesion of piliated *E. coli* to 1M-BSA- and BSA coated surfaces. The Ala188Asp amino acid substitution destabilizes the FimH$_L$ / FimH$_P$ domain interface and is a mimic for an increased population of the shear induced domain separated state $\mathbf{S_{bound}}$ [1,2]. (**a**) The mean speed of all bacteria within the time of observation is given. The speed for *E. coli* is significantly slowed on 1M-BSA compared to BSA-coated surface and reflects enhanced FimH dependent adhesion. Upon addition of 100 µM *n*-heptyl α-D-mannoside (HM) bacterial velocity on 1M-BSA is increased. (**b**) Distribution histogram of the mean speed

of tracks analyzed. (**c**) Fraction of tracked cells that are pre-attached (yellow bar)(<0.5 µm s^{-1}), permanently attached (red bar), mobile (white bar), transiently attached (yellow bar) or permanently detached (blue bar) within the observation. $FimH^{F18}$-piliated *E. coli* almost exclusively show transient attachment. $FimH^{F18}$-Ala188Asp piliated *E. coli* show less transient (7.2 %), but mainly permanent attachment (11.5 %) to 1M-BSA. Transient and permanent attachment to 1M-BSA is significantly reduced in the presence of HM. (**d**) In 1815 analyzed tracks of *E. coli* $FimH^{F18}$ on 1M-BSA 251 tracks (13.9 %) showed a transient attachment event: At the beginning of the observation the bacteria were mobile, then attached to 1M-BSA surface and after a duration of attachment of several seconds the bacteria detached from the surface and turn back swimming. The fraction of these cell tracks is plotted against the duration of attachment. The mean duration of transient attachment is 6.9 s. *E. coli* $FimH^{F18}$ attach mainly for a few seconds. In addition, the fraction of $FimH^{F18}$-Ala188Asp piliated bacteria that show a transient attachment event (88 out of 1'314 tracks) is plotted against the duration of attachment. The mean duration of attachment is 35.2 s, five times longer than for $FimH^{F18}$. (**e**) For $FimH^{F18}$ piliated bacteria on 1M-BSA (251 of 1'815 tracks) we found 67 tracks showing two transient attachment events (Phenotype: mobile, transient attachment, mobile, transient attachment, mobile) with a mean duration of attachment of 13.5 s.

Supplementary Notes

The newly designed probe ligand GN-FP-4 exhibits an about two-fold decrease in fluorescence at 520 nm (Supplementary Figs. 5a,b) and an about 7-fold increase in fluorescence polarization upon binding to isolated $FimH_L$ (Fig. 5a). Equilibrium fluorescence titration experiments revealed that GN-FP-4 binds with subnanomolar affinity to $FimH_L^{K12}$ and $FimH_L^{F18}$ (K_d values of 70 and 180 pM, respectively) (Supplementary Figs. 5d,e). With this 16-fold higher affinity compared to HM, GN-FP-4 is a promising reporter molecule for identifying high-affinity FimH binders via compound library screening. In an inverse competition experiment, in which HM in preformed $FimH_L$·HM complexes was displaced by GN-FP-4, the rate of HM displacement by GN-FP-4 proved to be first-order and independent of GN-FP-4 concentration (Supplementary Figs. 5f-j), demonstrating that HM dissociation was rate limiting for ligand exchange. Consequently, the observed rate in GN-FP-4 fluorescence decrease was identical to the rate of dissociation of HM from the isolated lectin domains. GN-FP-4 can thus also be used to screen FimH ligands for low off-rates.

Supplementary References

1. Yakovenko, O. et al. FimH forms catch bonds that are enhanced by mechanical force due to allosteric regulation. *J. Biol. Chem.* **283**, 11596-605 (2008).
2. Aprikian, P. et al. Interdomain interaction in the FimH adhesin of Escherichia coli regulates the affinity to mannose. *J. Biol. Chem.* **282**, 23437-46 (2007).
